# Postacute Sequelae Following Omicron COVID-19 in Patients With Cancer

**DOI:** 10.1001/jamanetworkopen.2026.4037

**Published:** 2026-03-31

**Authors:** Liang En Wee, Muhammad Ismail Bin Abdul Malek, Yong Yi Tan, Jue Tao Lim, Wei Chong Tan, Jinghao Nicholas Ngiam, Matilda Lee, Elise Kiat Yee Vong, Calvin J. Chiew, Russell Jingxian Li, Iain Bee Huat Tan, David Chien Lye, Kelvin Bryan Tan

**Affiliations:** 1 Communicable Diseases Agency, Singapore; 2 Duke-NUS Graduate Medical School, National University of Singapore, Singapore; 3 Department of Infectious Diseases, Singapore General Hospital, Singapore; 4 National Centre for Infectious Diseases, Singapore; 5 Lee Kong Chian School of Medicine, Nanyang Technological University, Singapore; 6 Division of Medical Oncology, National Cancer Centre Singapore, Singapore; 7 Division of Infectious Diseases, National University Hospital, Singapore; 8 Department of Haematology-Oncology, National University Cancer Institute, Singapore; 9 Department of Medical Oncology, Tan Tock Seng Hospital, Singapore; 10 Ministry of Health, Government of Singapore, Singapore; 11 Genome Institute of Singapore, Singapore; 12 Saw Swee Hock School of Public Health, National University of Singapore, Singapore; 13 Department of Infectious Diseases, Tan Tock Seng Hospital, Singapore; 14 Yong Loo Lin School of Medicine, National University of Singapore, Singapore

## Abstract

**Importance:**

Information on the burden of postacute sequelae of SARS-CoV-2 infection (or long COVID) in patients with cancer during endemicity is limited.

**Objective:**

To evaluate the risk of postacute diagnoses and/or symptoms compatible with long COVID in a population-based cohort of patients with cancer and high rates of vaccination and/or boosting who were infected during Omicron predominance compared with those with negative test results (hereinafter, noninfected patients). Results were additionally stratified by COVID-19 severity and receipt of therapeutics.

**Design, Setting, and Participants:**

This retrospective, population-based cohort study used health care claims databases to construct cohorts of adult patients with cancer in Singapore who were infected with SARS-CoV-2 during Omicron predominance (January 1 through December 31, 2022), and contemporaneous noninfected patients. Patients were followed up to 300 days from the index date and data were analyzed from February 1, 2022, through October 27, 2023.

**Exposure:**

SARS-CoV-2 infection.

**Main Outcomes and Measures:**

Competing risks regression (death as a competing risk), with overlap weights applied, was used to estimate risks of new-incident diagnoses and/or symptoms compatible with long COVID following SARS-CoV-2 infection in patients with cancer compared with noninfected patients. Risks of postacute sequelae following COVID-19 hospitalization in patients with cancer were further contrasted against influenza hospitalizations (January 1, 2017, to December 31, 2022).

**Results:**

A total of 76 807 patients with cancer were included in the analysis (48 279 [62.9%] female); 39 256 had SARS-CoV-2 infection and 37 551 were noninfected patients. The mean (SD) age was 63.9 (13.7) years. The mean (SD) follow-up time was 263.1 (36.2) days for patients infected with SARS-CoV-2 and 264.8 (32.5) days for noninfected patients. Most patients had solid-organ cancer (72 497 of 76 807 [94.4%]) and were boosted (71 550 of 76 807 [93.2%]); only a minority with SARS-CoV-2 infection (3571 of 39 256 [9.1%]) required acute hospitalization. No significant difference in risk of postacute diagnoses compatible with long COVID was observed in patients with SARS-CoV-2 infection (hazard ratio [HR], 0.98; 95% CI, 0.92-1.04) compared with noninfected patients. While risk of postacute symptoms following COVID-19 was modestly increased (HR, 1.09; 95% CI, 1.01-1.19; *P* = .048), statistical significance was not attained after adjustment for multiple comparisons. However, significantly increased risk of postacute sequelae was observed among patients hospitalized for COVID-19 compared with noninfected patients (HR for any diagnosis, 1.36 [95% CI, 1.18-1.56]; HR for any symptom, 1.48 [95% CI, 1.22-1.76]; *P* < .001 for both); risks remained elevated even among hospitalized cases receiving COVID-19 therapeutics. Risks of postacute sequelae following COVID-19 hospitalization in patients with cancer did not significantly differ from those associated with seasonal influenza hospitalizations.

**Conclusions and Relevance:**

The findings of this cohort study suggest that among highly boosted patients with cancer, the overall risk of postacute sequelae following Omicron SARS-CoV-2 infection was not significantly elevated compared with noninfected patients; however, patients who were hospitalized for COVID-19 remained at increased risk of postacute sequelae despite administration of COVID-19 therapeutics. These findings further suggest that COVID-19 vaccination and boosting remain important in mitigating the risk of long COVID among immunocompromised patients during endemicity.

## Key Points

**Question:**

What is the risk of postacute sequelae of SARS-CoV-2 infection (or long COVID) in patients with cancer who are vaccinated and/or boosted against COVID-19 and infected with SARS-CoV-2 during Omicron predominance?

**Findings:**

In a cohort of 76 807 patients with cancer, no significant elevation in overall risk of postacute sequelae compatible with long COVID was observed among those infected with the SARS-CoV-2 Omicron variant, compared with noninfected patients. However, patients with cancer who were hospitalized for COVID-19 had a 36% to 48% higher risk of postacute sequelae compared with noninfected patients.

**Meaning:**

These findings suggest that emergence of a milder Omicron variant and widespread vaccination attenuated risk of long COVID among patients with cancer during endemicity, except for hospitalized patients with more severe COVID-19.

## Teaser

This cohort study evaluates the risk of postacute diagnoses and/or symptoms compatible with long COVID among patients with cancer in Singapore who were infected with SARS-CoV-2 during the Omicron predominance compared with those who tested negative for COVID-19.

## Introduction

Immunocompromised individuals are more vulnerable to COVID-19; weakened immunity may delay SARS-CoV-2 clearance,^1^ with viral persistence linked to postacute sequelae of SARS-CoV-2 infection (or long COVID).^2^ Immunocompromised patients with cancer have increased long COVID risk vs non-immunocompromised individuals in large electronic health record studies^3,4^; but absence of a control group with negative test results limits estimates of population-wide prevalence.^5^ In a systematic review, the pooled prevalence of long COVID across several small prospective cohorts with cancer (N = 6653) was 20.5%, with at least 10% reporting long COVID symptoms as long as 12 months after infection^6^; but generalizability was significantly limited by small sample sizes and wide heterogeneity.

Previous prospective studies reporting long COVID sequelae in patients with cancer were conducted primarily during prevaccination pandemic waves, limiting current applicability.^7,8,9^ Emergence of milder Omicron variants and widespread vaccination may have altered long COVID risk.^10^ A multicenter registry of SARS-CoV-2–infected patients with cancer (N = 1909) recorded lower long COVID prevalence (6.2%) during Omicron predominance.^7,11^ In contrast, an elevated risk of long-term cardiovascular sequelae after COVID-19 was still observed in a large retrospective Omicron-infected cohort with cancer (N = 22 335); however, only one-third were boosted.^12^ We evaluated the risk of multisystemic postacute diagnoses and/or symptoms compatible with long COVID in a cohort with cancer and high rates of vaccination and boosting who became infected during Omicron predominance compared with those who tested negative for COVID-19 (hereinafter, noninfected patients). Results were additionally stratified based on initial severity and receipt of COVID-19 treatment.

## Methods

This cohort study was undertaken as national public health research under the Infectious Diseases Act of Singapore; accordingly, separate ethics review by an Institutional Review Board was not required, and a waiver of informed consent was granted because all data were deidentified. The study followed the Strengthening the Reporting of Observational Studies in Epidemiology (STROBE) reporting guideline.

### Study Population, Study Period, and Data Sources

This retrospective population-based cohort study used Singapore’s national COVID-19 testing database (population: 6.03 million) to identify cohorts of adult patients (aged ≥18 years) with cancer who were first infected with SARS-CoV-2 during Omicron-predominant transmission, as well as a contemporaneous cohort of noninfected patients with cancer. The enrollment period therefore encompassed January 1 through December 31, 2022, when SARS-CoV-2 testing (polymerase chain reaction and/or rapid antigen testing) was subsidized and widely available for all patients presenting with acute respiratory illness at any health care provider (ie, hospitals, public and private primary-care clinics).^13^ Notification of all SARS-CoV-2 tests performed in health care settings to our Ministry of Health was mandatory for all health care providers.^13^ Prior to the emergence of the Delta variant, community transmission was kept in check by extensive public health measures^13,14^; Omicron BA.1/2 replaced Delta in January 2022,^13^ followed by BA.4/5 in July 2022 and subsequently XBB in October 2022,^15^ resulting in COVID-19 surges during these periods (eFigure 1 in Supplement 1). Vaccination status was determined by National Immunisation Registry records. Two-dose primary vaccination was rolled out in 2020, with boosters introduced in September 2021 during the Delta surge.^14^

Risks of postacute diagnoses and/or symptoms compatible with long COVID- were assessed using the national health care claims database (Mediclaims), covering claims made against the national health savings and insurance schemes for inpatient or outpatient treatment at public and private health care providers in Singapore’s universal health care system. This database has been previously used to assess multisystemic risk of long COVID in the general adult Singaporean population.^16,17,18,19^

### Study Cohort

**Figure 1.**
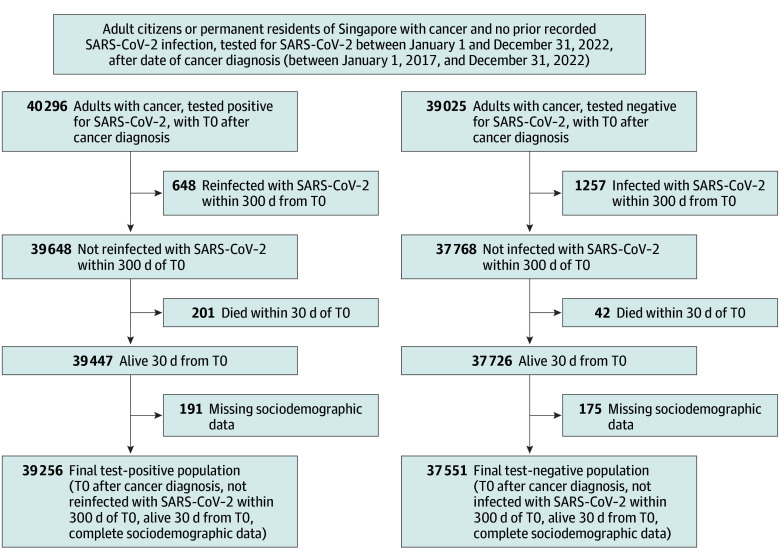
Study Flowchart The index date (T0) was taken as the date of test for both SARS-CoV-2–infected patients and those with negative test results.

A flowchart of the study cohort is provided in Figure 1. Patients with cancer who were first infected during Omicron-predominant transmission and died within 30 days of the index date (T0; date of test), who were reinfected within 300 days of T0_,_ or with missing sociodemographic data were additionally excluded. The noninfected patient group comprised test-negative patients with no record of COVID-19 prior to the enrollment period and a documented contemporaneous negative SARS-CoV-2 test result following cancer diagnosis. Test-negative patients who were infected within 300 days of T0, died within 30 days of T0, or had missing data were similarly excluded.

### Prespecified Outcomes

The primary outcome was any postacute diagnosis compatible with long COVID, defined as a composite of any new-incident cardiovascular, neurologic, psychiatric, autoimmune, respiratory, gastrointestinal, and/or kidney-related diagnoses, and any postacute symptom, defined as a composite of symptoms compatible with long COVID,^19,20^ at 31 to 300 days after T0. Individuals were therefore followed up to 300 days from T0, with the follow-up period encompassing February 1, 2022, to October 27, 2023. New-incident postacute diagnoses and/or symptoms were identified using *International Statistical Classification of Diseases, Tenth-Revision* (*ICD-10*), codes recorded in Mediclaims,^16,17,18,19,20^ minimizing selection bias caused by loss to follow-up via use of a comprehensive health care claims database with national-level coverage. For estimation of risks for each new-incident diagnosis or symptom, a subcohort of individuals without history of the studied diagnosis or symptom reported in the past 5 years in Mediclaims was constructed. Secondary outcomes included cardiovascular diagnoses,^16^ neurologic diagnoses,^17^ psychiatric diagnoses,^17^ autoimmune disorders (connective tissue disorders and/or vasculitis),^18^ respiratory sequelae, kidney impairment, and gastrointestinal diagnoses. Additionally, individual incidence of various common long COVID symptoms was evaluated, including cardiorespiratory symptoms, headache, joint pain, anosmia, memory loss, and fatigue or malaise, following prior methodology.^19,20^ Relevant definitions and *ICD-10* codes are detailed in the eAppendix in Supplement 1.

### Covariates

The following covariates were recorded from Ministry of Health databases: demographic characteristics (age, sex, ethnicity [categorized as Chinese, Indian, Malay, or other ethnicity], socioeconomic status by housing type),^14^ COVID-19 vaccination status (unboosted or boosted), comorbidities, and prior use of health care services (any prior hospitalization, emergency department visit in the year preceding T0, prior influenza vaccination). Ethnicity data were collected because ethnicity may be a risk factor for long COVID and COVID-19 severity. For cancer, type of cancer, time from cancer diagnosis to T0, and active cancer treatment (chemotherapy) were additionally obtained from Mediclaims.^21^

### Statistical Analysis

Data were analyzed from February 1, 2022, through October 27, 2023. Baseline characteristics were described using proportions and percentages for categorical data and means and SDs for continuous data. Standardized mean differences (SMDs) between groups were also computed, and between-group differences were accounted for using overlap weighting, where overlap weights were defined as 1 − propensity scores for COVID-19 cases and equivalent to the propensity scores for noninfected patients, thereby addressing the issue of extreme propensity scores by down-weighting patients in the tails of the propensity-score distribution.^22^ All individuals were therefore included in the weighted analysis, as patients with extreme propensity scores were down-weighted instead of being removed. All available covariates were incorporated in overlap weighting. An SMD less than 0.1 was taken as the threshold for good covariate balance after weighting. Hazard ratios (HRs) of new-incident multisystemic diagnoses and/or symptoms 31 to 300 days following T0 SARS-CoV-2 infection were estimated in patients with COVID-19 compared with noninfected patients using competing risks regression with overlap weights applied; death was taken as a competing risk. Individuals were followed up to 300 days after infection or to death, whichever occurred first. Risk trajectories of any postacute diagnosis or symptom in SARS-CoV-2–infected patients vs noninfected patients were estimated using the Kaplan-Meier approach, with overlap weights applied. Excess burden (EB) of new-incident multisystemic complications per 1000 individuals at 300 days of follow-up was defined as the increase or decrease in weighted-incidence rate in SARS-CoV-2–infected patients with cancer compared with noninfected patients. Patients with COVID-19 were further stratified by acute severity (hospitalized or nonhospitalized) and contrasted against all noninfected patients.

Subgroup analyses by age (<60 or ≥60 years), sex, vaccination status (unboosted or boosted), cancer type (solid organ or hematologic), time since cancer diagnosis, and concurrent active chemotherapy were performed; additionally, subgroup differences were evaluated for statistical significance through inclusion of an interaction term in regression analyses. To evaluate the impact of treatment on the risk of postacute sequelae in hospitalized patients with COVID-19 compared with noninfected patients, hospitalized COVID-19 cases were stratified by receipt of any antiviral (remdesivir, molnupiravir, or nirmatrelvir-ritonavir) or monoclonal antibody (sotrovimab or tixagevimab-cilgavimab) administered within 7 days of T0; receipt of COVID-19 therapeutics was tracked using Ministry of Health data.^19,23^

Several sensitivity analyses were performed. First, inverse probability weights were used as an alternative weighting scheme in place of overlap weights to estimate HRs, with use of the doubly robust approach, where covariates used to construct inverse probability weights were included as explanatory variables in the model specification. Second, unweighted Cox proportional hazards regression was performed as an alternative to weighting, including all specified covariates in regression models. Third, as rostered routine testing of asymptomatic individuals was largely discontinued from early February 2022, most COVID-19 tests in our cohort would have involved symptomatic individuals; nevertheless, enrollment was truncated to start only after official cessation of routine rostered testing in Singapore on April 1, 2022.^24,25^ Fourth, for patients with solid organ cancer and available staging information via linkage with the national Singapore Cancer Registry,^26^ cancer stage was additionally incorporated in weighting. Fifth, noninfected patients were additionally matched to patients with COVID-19 by T0 (calendar time) to ensure that T0 was balanced between groups. Sixth, risks of postacute sequelae following COVID-19 hospitalization were additionally contrasted against influenza hospitalizations (January 1, 2017, to December 31, 2022) in patients with cancer, contemporaneous noninfected patients with cancer hospitalized for non–SARS-CoV-2 viral pneumonia, and contemporaneous noninfected patients with cancer with a concurrent hospitalization (all-cause). Postacute sequelae have been similarly reported after hospitalization for non–SARS-CoV-2 respiratory viral infections^27,28,29,30^; however, extensive pandemic-related public-health measures limited contemporaneous influenza transmission.^31^ Seventh, risk of any postacute use of health care services (hospitalization or emergency department visit) 300 days after T0 in hospitalized or nonhospitalized SARS-CoV-2–infected patients with cancer vs comparators (contemporaneous noninfected patients or those with influenza hospitalizations) was evaluated as a separate outcome. Eighth, risks of postacute sequelae following COVID-19 hospitalization were contrasted against nonhospitalized SARS-CoV-2–infected patients to evaluate whether estimates differed from the main analysis. Ninth, incidence of atopic dermatitis or limb injuries in COVID-19 cases and noninfected patients was used as a negative-outcome control, given absence of causal associations between SARS-CoV-2 exposure and these conditions. To account for multiple comparisons, the threshold of statistical significance (Bonferonni correction) was set at 2-sided *P* < .005 for composite outcomes (n = 9) and 2-sided *P* < .002 for individual outcomes (n = 29), respectively. Analyses were conducted using Stata, version 16.0 (StataCorp LLC).

## Results

### Cohort Characteristics

During the study period, 40 296 cases of COVID-19 in patients with cancer were recorded; of these, 39 256 patients fulfilled inclusion criteria. In addition, 37 551 noninfected patients with cancer were enrolled, for a total of 76 807 patients in the overall cohort (48 279 [62.9%] female and 28 528 [37.1%] male; mean [SD] age, 63.9 [13.7] years) (Figure 1). The mean (SD) follow-up time in the SARS-CoV-2–infected group was 263.1 (36.2) days, while the noninfected patients were followed up for 264.8 (32.5) days from T0. Demographic and clinical characteristics are presented in Table 1. Most patients had solid organ cancer (72 497 [94.4%]) and were boosted (71 550 [93.2%]); only 3571 patients with COVID-19 (9.1%) were hospitalized. After weighting, demographic and clinical characteristics were balanced between groups, with all SMDs less than 0.01 (Table 1).

**Table 1.** Baseline Characteristics of SARS-CoV-2–Infected and Noninfected Patients With Cancer

Characteristic	Baseline, No. (%)		SMD at baseline	After weighting, No. (%)		SMD after weighting^a^
	SARS-CoV-2–infected patients (N = 39 256)	Noninfected patients (N = 37 551)		SARS-CoV-2–infected patients	Noninfected patients	
Age, mean (SD), y	63.9 (13.7)	64.1 (13.5)	0.02	63.9 (8.0)	63.9 (8.0)	<0.01
Age group, y						
18-39	2067 (5.3)	1858 (4.9)	0.01	966 (5.1)	966 (5.1)	<0.01
40-49	3926 (10.0)	3531 (9.4)	0.02	1831 (9.7)	1831 (9.7)	<0.01
50-59	7490 (19.1)	7131 (19.0)	0.00	3593 (19.1)	3593 (19.1)	<0.01
60-69	11 294 (28.8)	11 076 (29.5)	0.02	5492 (29.1)	5492 (29.1)	<0.01
70-79	9676 (24.6)	9560 (25.5)	0.02	4716 (25.0)	4716 (25.0)	<0.01
≥80	4803 (12.2)	4395 (11.7)	0.02	2246 (11.9)	2246 (11.9)	<0.01
Sex						
Female	24 486 (62.4)	23 793 (63.4)	0.02	11 856 (62.9)	11 856 (62.9)	<0.01
Male	14 770 (37.6)	13 758 (36.6)	0.02	6988 (37.1)	6988 (37.1)	<0.01
Ethnicity						
Chinese	32 966 (84.0)	31 637 (84.3)	0.01	15 879 (84.3)	15879 (84.3)	<0.01
Indian	2028 (5.2)	2210 (5.9)	0.03	1039 (5.5)	1039 (5.5)	<0.01
Malay	3566 (9.1)	2815 (7.5)	0.06	1546 (8.2)	1546 (8.2)	<0.01
Other^b^	696 (1.8)	889 (2.4)	0.04	380 (2.0)	380 (2.0)	<0.01
Housing type^c^						
1- to 2-Room public housing	1961 (5.0)	2061 (5.5)	0.02	986 (5.2)	986 (5.2)	<0.01
3- to 4-Room public housing	19 923 (50.8)	17 617 (46.9)	0.08	9221 (48.9)	9221 (48.9)	<0.01
5-Room (maximum) public housing	14 495 (36.9)	14 101 (37.6)	0.01	7036 (37.3)	7036 (37.3)	<0.01
Private housing or other	2877 (7.3)	3772 (10.0)	0.10	1601 (8.5)	1601 (8.5)	<0.01
Comorbidity burden^d^						
Concurrent cardiovascular disease	1293 (3.3)	989 (2.6)	0.04	552 (2.9)	552 (2.9)	<0.01
Concurrent cerebrovascular disease	2150 (5.5)	1743 (4.6)	0.04	943 (5.0)	943 (5.0)	<0.01
Concurrent chronic pulmonary disease	1961 (5.0)	1715 (4.6)	0.02	897 (4.8)	897 (4.8)	<0.01
Concurrent chronic liver disease	1160 (3.0)	955 (2.5)	0.03	512 (2.7)	512 (2.7)	<0.01
Concurrent chronic kidney disease	3109 (7.9)	2318 (6.2)	0.07	1309 (7.0)	1309 (7.0)	<0.01
Diabetes	9927 (25.3)	8341 (22.2)	0.07	4460 (23.7)	4460 (23.7)	<0.01
Type of cancer^e^						
Leukemia or lymphoma	2246 (5.7)	2064 (5.5)	0.01	1048 (5.6)	1048 (5.6)	<0.01
Breast	11 596 (29.5)	11 516 (30.7)	0.02	5678 (30.1)	5678 (30.1)	<0.01
Colorectal	5413 (13.8)	5220 (13.9)	0.00	2611 (13.9)	2611 (13.9)	<0.01
Lung	1694 (4.3)	1337 (3.6)	0.04	729 (3.9)	729 (3.9)	<0.01
Prostate	3415 (8.7)	3227 (8.6)	0.00	1633 (8.7)	1633 (8.7)	<0.01
Other solid organ	14 892 (37.9)	14 187 (37.8)	0.00	7145 (37.9)	7145 (37.9)	<0.01
Time between cancer and index date <1 y^f^	5035 (12.8)	4019 (10.7)	0.07	2184 (11.6)	2184 (11.6)	<0.01
Receiving active treatment for cancer (past 6 mo)	7133 (18.2)	5764 (15.3)	0.08	3126 (16.6)	3126 (16.6)	<0.01
Prior use of health care services						
No prior emergency department visit in the preceding 1 y	9890 (25.2)	7107 (18.9)	0.15	4077 (21.6)	4077 (21.6)	<0.01
No prior hospitalization in the preceding 1 y	18 535 (47.2)	16 166 (43.1)	0.08	8455 (44.9)	8455 (44.9)	<0.01
Influenza vaccination at index date	6758 (17.2)	6737 (17.2)	0.02	3317 (17.6)	3317 (17.6)	<0.01
COVID-19 vaccination status at index date						
Unvaccinated or partially vaccinated	650 (1.7)	578 (1.5)	0.01	302 (1.60)	302 (1.60)	<0.01
Fully vaccinated	2424 (6.2)	1605 (4.3)	0.09	955 (5.1)	955 (5.1)	<0.01
Boosted	22 570 (57.5)	19 708 (52.5)	0.10	10390 (55.1)	10390 (55.1)	<0.01
Doubly boosted	13 612 (34.7)	15 660 (41.7)	0.15	7197 (38.2)	7197 (38.2)	<0.01
SARS-CoV-2 subvariant in circulation at index date^g^						
Omicron BA1/2 or 4/5	33 510 (85.4)	32 547 (86.7)	0.04	16203 (86.0)	16203 (86.0)	<0.01
Omicron XBB subvariants	5746 (14.6)	5004 (13.3)	0.04	2642 (14.0)	2642 (14.0)	<0.01

Abbreviation: SMD, standardized mean difference.

^a^ Calculated after overlap weighting of SARS-CoV-2–infected patients and noninfected patients, weighted from original samples.

^b^ Includes other or multiple ethnicities.

^c^ Used as a surrogate marker of socioeconomic status.

^d^ Defined using comorbidities listed in the Charlson Comorbidity Index.

^e^ Cancer was defined as an *International Statistical Classification of Disease, Tenth Revision*, code C00.x-C41.x, C45.x-C97.x, recorded in the national health care claims database within 5 years from the index date. Skin cancer (eg, C43x and C44x) was excluded because of the relatively lower mortality of malignant nonmelanoma neoplasm of skin and its lower incidence in the predominantly Asian population in Singapore.

^f^ Index date was taken as date of positive SARS-CoV-2 test result in COVID-19 cases and date of negative SARS-CoV-2 test result in noninfected patients with cancer.

^g^ Predominant circulating SARS-CoV-2 variant at index date was determined based on national genomic surveillance data (≥90% of sequenced samples); Omicron BA.1/2 replaced Delta in January 2022, with a shift to BA.4/5 subvariants by June 2022. From October 2022 onward, various Omicron XBB subvariants predominated community transmission.

### Risk of Postacute Sequelae

Overall, the risk of postacute diagnoses both as a composite outcome and across multiple organ systems was not significantly increased in SARS-CoV-2–infected patients with cancer at 300 days post infection compared with noninfected patients (HR for any postacute diagnosis, 0.98; 95% CI, 0.92-1.04) (Table 2). Postacute symptoms showed a modest increase in risk and EB among SARS-CoV-2–infected patients with cancer (HR, 1.09 [95% CI, 1.01-1.19; *P* = .048]; EB per 1000 individuals, 2.66 [95% CI, 0.15-5.16]), although this difference did not remain significant after adjustment for multiple comparisons (Table 2). Risks for individual postacute sequelae did not significantly differ between SARS-CoV-2–infected patients with cancer and noninfected patients (eTable 1 in Supplement 1).

**Table 2.** Composite New-Incident Postacute Sequelae in SARS-CoV-2–Infected and Noninfected Adult Patients With Cancer^a^

Sequelae	No./total No. (%) of patients with cancer		HR (95% CI)^b^	EB weighted per 1000 individuals (95% CI)^c^
	SARS-CoV-2–infected patients	Noninfected patients		
**All patients with cancer and COVID-19**				
Any postacute diagnosis^d^	2320/27 635 (8.4)	2333/27 751 (8.4)	0.98 (0.92 to 1.04)	−1.79 (−6.42 to 2.84)
Any cardiovascular diagnosis	732/37 096 (2.0)	647/35 885 (1.8)	0.99 (0.89 to 1.10)	−0.15 (−2.12 to 1.82)
Any neurologic diagnosis	905/36 331 (2.5)	842/35 347 (2.4)	1.00 (0.91 to 1.10)	−0.04 (−2.29 to 2.22)
Any psychiatric diagnosis	157/38 483 (0.4)	134/36 804 (0.4)	1.08 (0.86 to 1.37)	0.31 (−0.57 to 1.20)
Any autoimmune diagnosis	54/39 151 (0.1)	44/37 467 (0.1)	1.14 (0.77 to 1.71)	0.17 (−0.34 to 0.68)
Any respiratory diagnosis	269/37 871 (0.7)	234/36 309 (0.6)	1.02 (0.85 to 1.21)	0.11 (−1.07 to 1.29)
Any kidney-related diagnosis	732/36 044 (2.0)	633/35 169 (1.8)	1.02 (0.91 to 1.13)	0.30 (−1.71 to 2.30)
Any gastrointestinal diagnosis	1161/34 988 (3.3)	1090/33 864 (3.2)	1.03 (0.94 to 1.11)	0.82 (−1.83 to 3.47)
Any postacute symptom^e^	1177/36 317 (3.2)	987/35 160 (2.8)	1.09 (1.01 to 1.19)	2.66 (0.15 to 5.16)
**Patients hospitalized for COVID-19^f^**				
Any postacute diagnosis^d^	273/1528 (17.9)	2333/27 751 (8.4)	1.36 (1.18 to 1.56)	42.52 (23.03 to 62.01)
Any cardiovascular diagnosis	172/3031 (5.7)	647/35 885 (1.8)	1.57 (1.29 to 1.90)	19.28 (10.99 to 27.57)
Any neurologic diagnosis	155/2856 (5.4)	842/35 347 (2.4)	1.36 (1.12 to 1.65)	13.70 (5.24 to 22.16)
Any psychiatric diagnosis	26/3459 (0.8)	134/36 804 (0.4)	1.54 (0.96 to 2.48)	2.50 (−0.39 to 5.38)
Any autoimmune diagnosis	7/3549 (0.2)	44/37 467 (0.1)	1.24 (0.50 to 3.10)	0.37 (−1.12 to 1.86)
Any respiratory diagnosis	41/3300 (1.2)	234/36 309 (0.6)	1.08 (0.74 to 1.56)	0.84 (−3.01 to 4.68)
Any kidney-related diagnosis	174/2793 (6.2)	633/35 169 (1.8)	1.48 (1.22 to 1.80)	18.38 (9.48 to 27.28)
Any gastrointestinal diagnosis	140/3000 (4.7)	1090/33 864 (3.2)	1.18 (0.96 to 1.44)	6.71 (−1.04 to 14.46)
Any postacute symptom^e^	197/2983 (6.6)	987/35 160 (2.8)	1.48 (1.24 to 1.76)	20.32 (11.26 to 29.38)
**Patients not requiring initial hospitalization**				
Any postacute diagnosis^d^	2047/26 107 (7.8)	2333/27 751 (8.4)	0.94 (0.89 to 1.00)	−4.40 (−9.03 to 0.22)
Any cardiovascular diagnosis	560/34 065 (1.6)	647/35 885 (1.8)	0.90 (0.81 to 1.01)	−1.71 (−3.65 to 0.22)
Any neurologic diagnosis	750/33 475 (2.2)	842/35 347 (2.4)	0.96 (0.87 to 1.05)	−1.04 (−3.30 to 1.21)
Any psychiatric diagnosis	131/35 024 (0.4)	134/36 804 (0.4)	1.04 (0.82 to 1.33)	0.15 (−0.75 to 1.04)
Any autoimmune diagnosis	47/35 602 (0.1)	44/37 467 (0.1)	1.13 (0.75 to 1.71)	0.15 (−0.36 to 0.67)
Any respiratory diagnosis	228/34 571 (0.7)	234/36 309 (0.6)	1.00 (0.83 to 1.20)	0.02 (−1.17 to 1.20)
Any kidney-related diagnosis	558/33 251 (1.7)	633/35 169 (1.8)	0.93 (0.83 to 1.04)	−1.29 (−3.25 to 0.67)
Any gastrointestinal diagnosis	1021/31 988 (3.2)	1090/33 864 (3.2)	1.01 (0.93 to 1.10)	0.30 (−2.39 to 2.99)
Any postacute symptom^e^	980/33 334 (2.9)	987/35 160 (2.8)	1.05 (0.96 to 1.14)	1.34 (−1.17 to 3.85)

Abbreviations: EB, excess burden; HR, hazard ratio.

^a^ Numbers in each subcohort do not add up to the original number of SARS-CoV-2–infected patients or noninfected patients because for estimation of risks for each new-incident diagnosis, a subcohort of individuals without history of the diagnosis in the past 5 years was constructed.

^b^ Calculated using competing risks regression taking death as a competing risk, with overlap weights used. For patients with cancer, overlap was weighted based on demographic characteristics (age, sex, and ethnicity), socioeconomic status (housing type), COVID-19 vaccination status, comorbidities, prior use of health care services, prior influenza vaccination, type of cancer, time from cancer diagnosis to index date, receipt of active cancer treatment, and prevailing SARS-CoV-2 variant in circulation.

^c^ Defined as increase or decrease in incidence rate of new-incident sequelae among SARS-CoV-2–infected patients and noninfected patients. EB greater than 0 denotes EB of a respective composite or individual outcome among SARS-CoV-2–infected patients and noninfected patients.

^d^ Taken as a composite of any cardiovascular, neurologic, psychiatric, autoimmune, respiratory, kidney-related, or gastrointestinal diagnoses in patients with cancer.

^e^ Taken as a composite of any individual postacute symptoms, including cardiovascular signs or symptoms, respiratory signs or symptoms, headache, musculoskeletal pain or stiffness, abdominal or pelvic pain, generalized pain, loss of smell or taste, memory and cognitive impairment, fatigue, and malaise.

^f^ Defined as any hospitalization attributed to COVID-19, reported in the national COVID-19 database.

**Figure 2.**
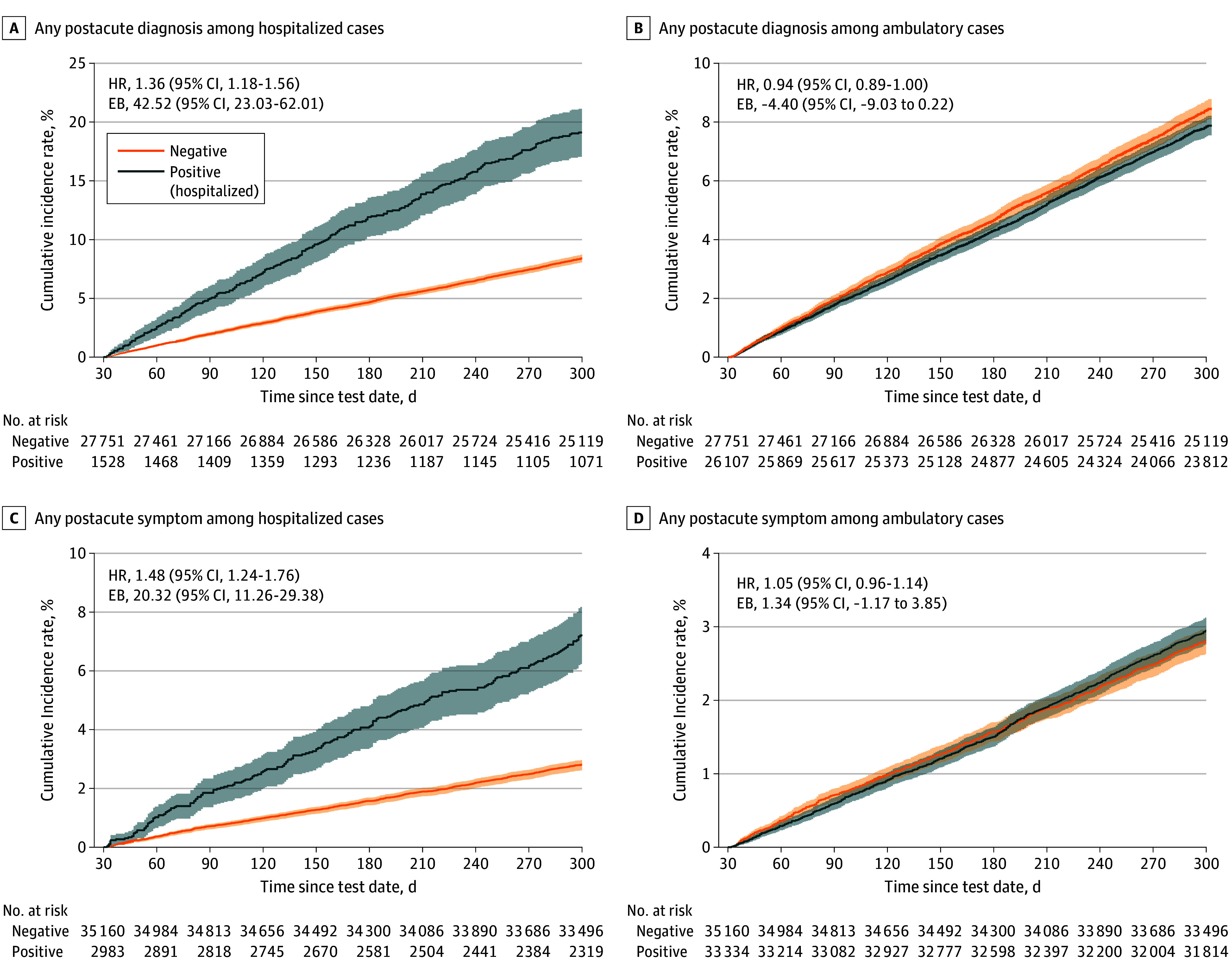
Survival Plots of Risks of Long COVID Diagnoses and Symptoms at 31 to 300 Days Following SARS-CoV-2 Omicron Infection in Patients With Cancer Patients with COVID-19 are stratified by infection severity (hospitalized and ambulatory). Solid lines represent estimated 31- to 300-day outcome probabilities of long COVID diagnoses and symptoms between patients with cancer who had COVID-19 and those who were not infected; shaded areas represent 95% CIs for those estimates. Risk trajectories were estimated using the Kaplan-Meier approach, with inverse propensity weights applied. EB indicates excess burden; HR, hazard ratio.

However, significantly increased risk and/or EB of postacute sequelae was observed among patients with cancer acutely hospitalized for COVID-19 compared with noninfected patients for any diagnosis (HR, 1.36 [95% CI, 1.18-1.56; *P* < .001]; EB per 1000 individuals, 42.52 [95% CI, 23.03-62.01]) or any symptom (HR, 1.48 [95% CI, 1.24-1.76; *P* < .001], EB per 1000 individuals, 20.32 [95% CI, 11.26-29.38]) (Table 2). In particular, increased risk and/or EB of postacute cardiovascular sequelae was observed among patients with cancer who were hospitalized for COVID-19 compared with noninfected patients (HR, 1.57 [95% CI, 1.29-1.90; *P* < .001]; EB per 1000 individuals, 19.28 [95% CI, 10.99-27.57]). Similarly, increased risk and/or EB of postacute kidney dysfunction was observed among patients with cancer who were hospitalized for COVID-19 compared with noninfected patients (HR, 1.48 [95% CI, 1.22-1.80; *P* < .001]; EB per 1000 individuals, 18.38 [95% CI, 9.48-27.28]). Risk trajectories for any postacute diagnosis or symptom in hospitalized COVID-19 patients compared with noninfected patients significantly diverged during the postacute period (Figure 2). Risks of postacute sequelae remained elevated even among hospitalized patients receiving COVID-19 therapeutics (HR for any diagnosis, 1.37 [95% CI, 1.13-1.66; *P* < .001]; HR for any symptoms, 1.57 [95% CI, 1.25-1.98; *P* < .001]) (eTable 2 in Supplement 1). In contrast, the risk of postacute sequelae in patients with cancer who had mild COVID-19 did not significantly differ from that of noninfected patients (Table 2), and risk trajectories for any postacute diagnosis or symptom in nonhospitalized patients with COVID-19 compared with noninfected patients converged (Figure 2).

### Subgroup and Sensitivity Analyses

Across subgroups, results of tests for interaction were not statistically significant. In exploratory analyses, risks of postacute sequelae did not significantly differ between patients infected with SARS-CoV-2 and noninfected patients when stratified by age, sex, COVID-19 vaccination, cancer type, time elapsed since cancer diagnosis, and active chemotherapy, except during Omicron XBB predominance, in which risks and/or EB of any postacute symptom were significantly increased in cases with COVID-19 compared with noninfected patients (HR, 1.33 [95% CI, 1.06-1.66; *P* < .001]; EB per 1000 individuals, 8.70 [95% CI, 1.85-15.55]) (Table 3). In sensitivity analyses, using alternative weighting schemes (doubly robust approach with inverse propensity weights [eTable 3 in Supplement 1]) or unweighted competing risks regression with the same variables included as covariates (eTable 4 in Supplement 1) did not significantly influence estimates. When enrollment was truncated to commence only after complete cessation of routine rostered testing nationally, similarly overall risk of postacute sequelae did not differ significantly in SARS-CoV-2–infected patients with cancer compared with noninfected patients, except for the subset requiring acute hospitalization for COVID-19 (eTable 5 in Supplement 1). Inclusion of cancer stage in weighting as a proxy for severity among the subset of patients with solid organ cancer and available staging data (42 782 of 72 497 [59.0%]) did not substantially change risk estimates; apart from the subset of patients hospitalized with COVID-19, overall risk of postacute sequelae did not differ significantly between patients with positive and negative test results, with the exception of postacute symptoms (HR, 1.32; 95% CI, 1.15-1.52; *P* < .001) (eTable 6 in Supplement 1). Noninfected patients were additionally matched to those with positive test results by T0, ensuring that T0 was similarly distributed between groups (eFigure 2 in Supplement 1), but this did not significantly change risk estimates (eTable 7 in Supplement 1). Risks of postacute sequelae following COVID-19 hospitalization did not significantly differ from those following hospitalizations for seasonal influenza or contemporaneous hospitalizations for viral pneumonia among noninfected patients with cancer (eTable 8 in Supplement 1), although elevated risk was observed after COVID-19 hospitalization compared with concurrently hospitalized noninfected patients (all-cause). Significantly increased risk of EB of postacute sequelae was still observed after COVID-19 hospitalization when nonhospitalized patients with COVID-19 were designated as the comparator (eTable 9 in Supplement 1). Postacute all-cause use of health care services to 300 days after T0 in SARS-CoV-2–infected patients with cancer not requiring initial hospitalization compared with noninfected patients did not significantly differ (eTable 10 in Supplement 1), although the minority of hospitalized patients with COVID-19 had higher risk of postacute use of health care services compared with all noninfected patients and previous influenza hospitalizations. Risks among noninfected patients for negative outcomes did not significantly differ between patients with cancer who were infected with SARS-CoV-2 and noninfected patients (HR for atopic dermatitis, 0.73 [95% CI, 0.39-1.36]; HR for upper-limb injuries, 0.94 [95% CI, 0.77-1.14]; HR for lower-limb injuries, 1.05 [95% CI, 0.88-1.26]) (eTable 11 in Supplement 1).

**Table 3.** Composite New-Incident Postacute Sequelae in SARS-CoV-2–Infected and Noninfected Patients With Cancer by Subgroup^a^

Subgroup^b^	No./total No. (%) of patients with cancer		HR (95% CI)^c^	EB, weighted, per 1000 individuals (95% CI)^d^
	SARS-CoV-2–infected patients	Noninfected patients		
**Age <60 y**				
Any postacute diagnosis	509/11 136 (4.6)	516/10 546 (4.9)	0.90 (0.80 to 1.02)	−4.59 (−10.24 to 1.07)
Any postacute symptom	256/12 653 (2.0)	200/11 866 (1.7)	1.10 (0.92 to 1.33)	1.84 (−1.52 to 5.21)
**Age ≥60 y**				
Any postacute diagnosis	1811/16 499 (11.0)	1817/17 205 (10.6)	1.00 (0.94 to 1.07)	−0.10 (−6.73 to 6.53)
Any postacute symptom	921/23 664 (3.9)	787/23 294 (3.4)	1.09 (0.99 to 1.20)	3.06 (−0.32 to 6.44)
**Females**				
Any postacute diagnosis	1294/18 579 (7.0)	1350/18 563 (7.3)	0.95 (0.88 to 1.02)	−3.79 (−9.02 to 1.45)
Any postacute symptom	681/22 820 (3.0)	593/22 401 (2.6)	1.08 (0.97 to 1.21)	2.27 (−0.78 to 5.31)
**Males**				
Any postacute diagnosis	1026/9056 (11.3)	983/9188 (10.7)	1.02 (0.94 to 1.12)	2.27 (−6.83 to 11.37)
Any postacute symptom	496/13 497 (3.7)	394/12 759 (3.1)	1.11 (0.97 to 1.26)	3.35 (−1.01 to 7.71)
**Did not receive COVID-19 booster**				
Any postacute diagnosis	181/1960 (9.2)	150/1486 (10.1)	0.91 (0.73 to 1.13)	−9.72 (−29.81 to 10.36)
Any postacute symptom	114/2752 (4.1)	71/1987 (3.6)	1.06 (0.79 to 1.44)	2.35 (−8.75 to 13.44)
**Received single COVID-19 booster**				
Any postacute diagnosis	1270/16 481 (7.7)	1174/14 983 (7.8)	0.95 (0.87 to 1.02)	−4.09 (−10.01 to 1.83)
Any postacute symptom	653/20 908 (3.1)	500/18 495 (2.7)	1.09 (0.97 to 1.23)	2.51 (−0.80 to 5.83)
**Received ≥2 COVID-19 booster doses**				
Any postacute diagnosis	869/9194 (9.5)	1009/11 282 (8.9)	1.03 (0.94 to 1.13)	2.82 (−5.15 to 10.80)
Any postacute symptom	410/12 657 (3.2)	416/14 678 (2.8)	1.10 (0.96 to 1.26)	2.78 (−1.31 to 6.86)
**Tested before Omicron XBB predominance^e^**				
Any postacute diagnosis	1956/23 649 (8.3)	2014/24 141 (8.3)	0.97 (0.91 to 1.03)	−2.67 (−7.63 to 2.29)
Any postacute symptom	983/31 048 (3.2)	860/30 483 (2.8)	1.06 (0.96 to 1.16)	1.67 (−1.02 to 4.36)
**Tested during Omicron XBB predominance^e^**				
Any postacute diagnosis	364/3986 (9.1)	319/3610 (8.8)	1.03 (0.89 to 1.20)	3.14 (−9.70 to 15.97)
Any postacute symptom	194/5269 (3.7)	127/4677 (2.7)	1.33 (1.06 to 1.66)	8.70 (1.85 to 15.55)
**Diagnosed with cancer <1 y from index date^f^**				
Any postacute diagnosis	328/3286 (10.0)	260/2715 (9.6)	1.01 (0.86 to 1.19)	0.79 (−14.36 to 15.94)
Any postacute symptom	173/4552 (3.8)	134/3672 (3.6)	0.98 (0.78 to 1.23)	−0.74 (−8.95 to 7.47)
**Diagnosed with cancer ≥1 y from index date^f^**				
Any postacute diagnosis	1992/24 349 (8.2)	2073/25 036 (8.3)	0.97 (0.91 to 1.04)	−2.16 (−7.01 to 2.70)
Any postacute symptom	1004/31 765 (3.2)	853/31 488 (2.7)	1.11 (1.01 to 1.22)	3.08 (0.46 to 5.70)
**Solid organ cancer**				
Any postacute diagnosis^d^	2192/26 079 (8.4)	2198/26 238 (8.4)	0.98 (0.93 to 1.04)	−1.47 (−6.23 to 3.29)
Any postacute symptom	1114/34 285 (3.2)	934/33 239 (2.8)	1.10 (1.00 to 1.20)	2.72 (0.14 to 5.30)
**Hematological cancer**				
Any postacute diagnosis	128/1556 (8.2)	135/1513 (8.9)	0.91 (0.71 to 1.16)	−7.56 (−27.38 to 12.25)
Any postacute symptom	63/2032 (3.1)	53/1921 (2.8)	1.06 (0.73 to 1.54)	1.68 (−8.76 to 12.12)
**Not receiving active treatment for cancer (past 6 mo)**				
Any postacute diagnosis	1909/22 561 (8.5)	1964/23 555 (8.3)	1.00 (0.94 to 1.06)	−0.17 (−5.23 to 4.90)
Any postacute symptom	928/29 906 (3.1)	806/29 858 (2.7)	1.10 (1.00 to 1.21)	2.62 (−0.07 to 5.30)
**Receiving active treatment for cancer (past 6 mo)**				
Any postacute diagnosis	411/5074 (8.1)	369/4196 (8.8)	0.89 (0.77 to 1.02)	−9.73 (−21.18 to 1.71)
Any postacute symptom	249/6411 (3.9)	181/5302 (3.4)	1.08 (0.89 to 1.31)	2.86 (−3.95 to 9.67)

Abbreviations: EB, excess burden; HR, hazard ratio.

^a^ Numbers in each subcohort do not add up to the original number of SARS-CoV-2–infected patients or noninfected patients because for estimation of risks for each new-incident diagnosis/symptom, a subcohort of individuals without history of the diagnosis or symptom in the past 5 years was constructed.

^b^ Any postacute diagnosis was taken as a composite of any cardiovascular, neurologic, psychiatric, autoimmune, respiratory, kidney-related, or gastrointestinal diagnoses. Any postacute symptom was taken as a composite of any individual postacute symptoms, including cardiovascular signs or symptoms, respiratory signs or symptoms, headache, musculoskeletal pain or stiffness, abdominal or pelvic pain, generalized pain, loss of smell or taste, memory and cognitive impairment, fatigue, and malaise.

^c^ Calculated using competing risks regression taking death as a competing risk, with overlap weights used. Regression weighted based on demographic characteristics (age, sex, and ethnicity), socioeconomic status (housing type), COVID-19 vaccination status, comorbidities, prior use of health care services, influenza vaccination, type of cancer, time from cancer diagnosis to index date, receiving active cancer treatment, and prevailing SARS-CoV-2 variant in circulation.

^d^ Defined as increase or decrease in incidence rate of new-incident sequelae among COVID-19 cases and controls in individuals with cancer. EB greater than 0 denotes EB of a respective composite or individual outcome among SARS-CoV-2–infected patients and noninfected patients.

^e^ Indicates predominant circulating SARS-CoV-2 variant at index date, determined based on national genomic surveillance data (≥90% of sequenced samples). Omicron BA.1/2 replaced Delta in January 2022, with a shift to BA.4/5 subvariants by June 2022. From October 2022 onward, various Omicron XBB subvariants predominated community transmission.

^f^ Index date was taken as date of positive SARS-CoV-2 test result in COVID-19 cases and date of negative SARS-CoV-2 test result in controls.

## Discussion

In this predominantly boosted cohort of patients with cancer, no significant elevation in overall risk of postacute sequelae was observed in patients up to 300 days following predominantly mild Omicron infection compared with noninfected patients. While patients with cancer who were acutely hospitalized for COVID-19 had a 36% to 48% increased risk of postacute sequelae compared with noninfected patients, the overall incidence was modest, and risks did not significantly differ from those following hospitalization for seasonal influenza.

Our findings of no difference in postacute sequelae following predominantly mild SARS-CoV-2 Omicron infection in boosted patients with cancer compared with noninfected patients differ from those of prior studies in the general adult Singaporean population, in which increased risk and/or EB of cardiovascular or neurologic sequelae was reported among adult survivors of Omicron COVID-19 compared with noninfected patients.^16,17^ In other prospective cohorts of patients with cancer who were predominantly infected before Omicron predominance, 15% to 33% reported long COVID,^6,7,8,9^ although differences in methodology and definitions precluded direct comparison. Prior cancer was associated with slower resolution of long COVID symptoms, although infections occurred in unvaccinated individuals and were attributed to ancestral SARS-CoV-2 variants.^32^ Given that long COVID has been attributed to postinfectious immune dysregulation,^33^ long COVID may paradoxically be less common in immunosuppressed patients with cancer due to lower risk of persistent postinfectious immune activation. Similarly, overall risk of postacute sequelae was not significantly elevated in a population-based cohort with immunosuppression (N = 3372) that enrolled roughly equal numbers of transplant recipients with Omicron SARS-CoV-2 infection and noninfected patients.^34^ In a small prospective cohort of SARS-CoV-2–infected patients with blood cancer (N = 94),^35^ long COVID burden reduced substantially over time, declining from 46% (ancestral strains) to 14% during Omicron predominance. While studies have suggested viral persistence as a potential long COVID mechanism,^2^ and immunosuppression may delay viral clearance, prolonged replication-competent Omicron SARS-CoV-2 infections are relatively uncommon.^36^ Although higher risk of postacute cardiovascular sequelae was observed in Omicron-infected vs matched noninfected patients with cancer in a large population-based Hong Kong cohort (N = 22 335), one-third were unvaccinated or partially vaccinated^12^; in comparison, at least 90% of our cohort were boosted. Our findings may therefore reflect differences arising from attenuated COVID-19 severity, attributed to vaccination and boosting.

Boosting and vaccination confer protection against severe COVID-19, even in immunocompromised patients. While lower immunogenicity was observed among patients with cancer vs immunocompetent individuals, most vaccinated patients with cancer still mounted robust cellular and humoral responses to SARS-CoV-2 in in vitro studies,^37^ with a vaccine booster increasing Omicron-specific neutralization among immunocompromised patients.^38^ No significantly increased risk of postacute sequelae among SARS-CoV-2–infected patients with cancer compared with noninfected patients was observed in this highly vaccinated and boosted cohort. In the general adult population, boosting has been associated with greater attenuation of risk for postacute sequelae.^10^ Infections recorded during Omicron XBB predominance had increased risks and/or EB of any postacute symptom compared with noninfected patients; this may reflect the more immune-evasive nature of newer SARS-CoV-2 variants, moderating the protection afforded by vaccination with ancestral vaccines. Continued monitoring for long COVID in patients with cancer infected with newer SARS-CoV-2 variants is warranted during COVID-19 endemicity.

Significantly increased risk and/or EB of postacute sequelae was observed among patients with cancer who were acutely hospitalized for COVID-19 compared with noninfected patients and when contrasted against mildly infected COVID-19 cases. In the general population, risk for long COVID increased in tandem with acute COVID-19 severity.^16,17,18^ However, postacute sequelae in hospitalized COVID-19 cases during Omicron predominance was comparable with that arising from seasonal influenza hospitalization or contemporaneous test-negative hospitalizations for viral pneumonia, highlighting risk for long-term sequelae following severe respiratory viral infection in general,^27,28,29,30^ including other vaccine-preventable infections.^39^

Persistent vaccine hesitancy among patients with cancer during endemicity therefore represents a missed preventative opportunity,^40,41,42,43^ given the significant clinical and economic burden of COVID-19 among immunocompromised individuals.^44^ Risk of postacute sequelae remained elevated even among patients with COVID-19 who received treatment. In contrast, lower postacute risk of all-cause mortality was observed among nirmatrelvir-ritonavir–treated individuals in an immunocompromised Hong Kong cohort, although effectiveness was less pronounced in immunocompromised vs immunocompetent individuals.^45^ Higher uptake of vaccination and boosting may have mitigated acute COVID-19 severity in our population and thereby made protection afforded by nirmatrelvir-ritonavir against postacute sequelae more marginal; antiviral treatment (remdesivir or nirmatrelvir-ritonavir) did not significantly reduce the risk of postacute multisystemic sequelae among boosted older individuals in Singapore.^19,23^

### Limitations

Our study has some limitations. Misclassification remains possible due to unreported asymptomatic or mild infections. Serology was not routinely tested, and national COVID-19 databases only included results of all SARS-CoV-2 tests performed in health care settings but not results of self-administered home testing with rapid antigen tests. Although risks of postacute sequelae were examined using a comprehensive health care claims database with national-level coverage, claims-based methodology may underestimate milder sequelae not affecting reimbursement, biasing estimates toward the null. Direct immunologic and virologic data and clinical confirmation of postacute sequelae as potentially attributable to SARS-CoV-2 infection were unavailable in this retrospective study; our findings are therefore exploratory and require further mechanistic validation in prospective clinical cohorts. The observed higher risk of postacute sequelae in hospitalized patients should be interpreted cautiously due to potential surveillance bias from increased monitoring, selection bias from baseline disease severity, and attribution bias in distinguishing cancer symptoms from long COVID manifestations. Information on cancer severity (eg, staging) was not comprehensively recorded in national health care claims data, and limited granularity on chemotherapy meant that risks in subgroups on more immunosuppressive regimens (eg, B-cell–depleting therapies) could not be ascertained. Last, our findings from this highly vaccinated population may have limited generalizability to settings with lower vaccination coverage.

## Conclusions

In this cohort study of 76 807 patients with cancer, at least 90% of whom were boosted, the overall risk of postacute sequelae 300 days following Omicron SARS-CoV-2 infection was not significantly elevated compared with noninfected patients. However, patients with cancer who were hospitalized for COVID-19 remained at increased risk of postacute sequelae, even among treated individuals. These findings suggest that COVID-19 vaccination and boosting remain important in mitigating long COVID risk among immunocompromised patients with cancer during endemicity.

## Notes

Supplement 1. **eTable 1. **Hazards Ratio (HR) of Individual New-Incident Postacute Sequelae in SARS-CoV-2–Infected Adult Patients With Cancer and Noninfected Controls **eTable 2. **Hazards Ratio (HR) of Composite New-Incident Postacute Sequelae in Patients With Cancer Hospitalized for COVID-19, Stratified by Receipt of COVID-19 Treatment vs Noninfected Controls **eTable 3.** Hazards Ratio (HR) of Composite New-Incident Postacute Sequelae in SARS-CoV-2–Infected Patients With Cancer and Noninfected Controls, Using Alternative Weighting Scheme (Inverse Propensity Weighting) **eTable 4. **Adjusted Hazards Ratio (AHR) of Composite New-Incident Postacute Sequelae in SARS-CoV-2–Infected Patients With Cancer and Noninfected Controls Using Competing Risks Regression (Unweighted) **eTable 5. **Adjusted Hazards Ratio (AHR) of Composite New-Incident Postacute Sequelae in SARS-CoV-2–Infected Patients With Cancer and Noninfected Controls After Cessation of Routine Rostered Testing for Asymptomatic Individuals **eTable 6. **Adjusted Hazards Ratio (AHR) of Composite New-Incident Postacute Sequelae in SARS-CoV-2–Infected Patients With Cancer and Noninfected Controls in Subset of Solid Organ Cancer Cases With Staging Data Available **eTable 7. **Adjusted Hazards Ratio (AHR) of Composite New-Incident Postacute Sequelae in SARS-CoV-2–Infected Patients With Cancer and Noninfected Controls Matched by Time 0 **eTable 8. **Hazards Ratio (HR) of Composite New-Incident Postacute Sequelae in Patients With Cancer Hospitalized for COVID-19 vs Influenza Hospitalizations, Noninfected Controls Hospitalized for Respiratory Complications, and Noninfected Controls Hospitalized for Any Cause **eTable 9. **Hazards Ratio (HR) of Composite New-Incident Postacute Sequelae in Hospitalized vs Nonhospitalized SARS-CoV-2–Infected Patients With Cancer **eTable 10. **Hazards Ratio (HR) of Postacute Use of Health Care Resources (All-Cause) in SARS-CoV-2–Infected Patients With Cancer and Comparators **eTable 11. **Hazards Ratio (HR) of Negative Outcome Controls (Atopic Dermatitis, Limb Injuries) **eFigure 1. **Distribution of Test Date in SARS-CoV-2–Infected and Noninfected Patients With Cancer **eFigure 2. **Distribution of Test Date in SARS-CoV-2–Infected and Noninfected Patients With Cancer (After Matching by Test Date) **eAppendix. **List of *ICD-10* Codes Used for Diagnoses of Interest

Supplement 2.
Data Sharing Statement
