# Supplementary material for: Postacute Sequelae Following Omicron COVID-19 in Patients With Cancer
Source: JAMA Netw Open. 2026 Mar 31;9(3):e264037. doi: 10.1001/jamanetworkopen.2026.4037 (PMC13040405; doi:10.1001/jamanetworkopen.2026.4037)
Supplement: Supplement 1. — eTable 1. Hazards Ratio (HR) of Individual New-Incident Postacute Sequelae in SARS-CoV-2–Infected Adult Patients With Cancer and Noninfected Controls eTable 2. Hazards Ratio (HR) of Composite New-Incident Postacute Sequelae in Patients With Cancer Hospitalized for COVID-19, Stratified by Receipt of COVID-19 Treatment vs Noninfected Controls eTable 3. Hazards Ratio (HR) of Composite New-Incident Postacute Sequelae in SARS-CoV-2–Infected Patients With Cancer and Noninfected Controls, Using Alternative Weighting Scheme (Inverse Propensity Weighting) eTable 4. Adjusted Hazards Ratio (AHR) of Composite New-Incident Postacute Sequelae in SARS-CoV-2–Infected Patients With Cancer and Noninfected Controls Using Competing Risks Regression (Unweighted) eTable 5. Adjusted Hazards Ratio (AHR) of Composite New-Incident Postacute Sequelae in SARS-CoV-2–Infected Patients With Cancer and Noninfected Controls After Cessation of Routine Rostered Testing for Asymptomatic Individuals eTable 6. Adjusted Hazards Ratio (AHR) of Composite New-Incident Postacute Sequelae in SARS-CoV-2–Infected Patients With Cancer and Noninfected Controls in Subset of Solid Organ Cancer Cases With Staging Data Available eTable 7. Adjusted Hazards Ratio (AHR) of Composite New-Incident Postacute Sequelae in SARS-CoV-2–Infected Patients With Cancer and Noninfected Controls Matched by Time 0 eTable 8. Hazards Ratio (HR) of Composite New-Incident Postacute Sequelae in Patients With Cancer Hospitalized for COVID-19 vs Influenza Hospitalizations, Noninfected Controls Hospitalized for Respiratory Complications, and Noninfected Controls Hospitalized for Any Cause eTable 9. Hazards Ratio (HR) of Composite New-Incident Postacute Sequelae in Hospitalized vs Nonhospitalized SARS-CoV-2–Infected Patients With Cancer eTable 10. Hazards Ratio (HR) of Postacute Use of Health Care Resources (All-Cause) in SARS-CoV-2–Infected Patients With Cancer and Comparators eTable 11. Hazards Ratio (HR) of Negative Outcome Controls (Atopic [file jamanetwopen-e264037-s001.pdf]

Supplementary Online Content

Wee LE, Malek MIBA, Tan YY, et al. Postacute sequelae following Omicron COVID-19 in patients with cancer. *JAMA Netw Open.* 2026;9(3):e264037. doi:10.1001/jamanetworkopen.2026.4037

eTable 1. Hazards Ratio (HR) of Individual New-Incident Postacute Sequelae in SARS-CoV-2–Infected Adult Patients With Cancer and Noninfected Controls

eTable 2. Hazards Ratio (HR) of Composite New-Incident Postacute Sequelae in Patients With Cancer Hospitalized for COVID-19, Stratified by Receipt of COVID-19 Treatment vs Noninfected Controls

eTable 3. Hazards Ratio (HR) of Composite New-Incident Postacute Sequelae in SARS-CoV-2–Infected Patients With Cancer and Noninfected Controls, Using Alternative Weighting Scheme (Inverse Propensity Weighting)

eTable 4. Adjusted Hazards Ratio (AHR) of Composite New-Incident Postacute Sequelae in SARS-CoV-2–Infected Patients With Cancer and Noninfected Controls Using Competing Risks Regression (Unweighted)

eTable 5. Adjusted Hazards Ratio (AHR) of Composite New-Incident Postacute Sequelae in SARS-CoV-2–Infected Patients With Cancer and Noninfected Controls After Cessation of Routine Rostered Testing for Asymptomatic Individuals

eTable 6. Adjusted Hazards Ratio (AHR) of Composite New-Incident Postacute Sequelae in SARS-CoV-2–Infected Patients With Cancer and Noninfected Controls in Subset of Solid Organ Cancer Cases With Staging Data Available

eTable 7. Adjusted Hazards Ratio (AHR) of Composite New-Incident Postacute Sequelae in SARS-CoV-2–Infected Patients With Cancer and Noninfected Controls Matched by Time 0

eTable 8. Hazards Ratio (HR) of Composite New-Incident Postacute Sequelae in Patients With Cancer Hospitalized for COVID-19 vs Influenza Hospitalizations, Noninfected Controls Hospitalized for Respiratory Complications, and Noninfected Controls Hospitalized for Any Cause

eTable 9. Hazards Ratio (HR) of Composite New-Incident Postacute Sequelae in Hospitalized vs Nonhospitalized SARS-CoV-2–Infected Patients With Cancer

eTable 10. Hazards Ratio (HR) of Postacute Use of Health Care Resources (All-Cause) in SARS-CoV-2–Infected Patients With Cancer and Comparators

eTable 11. Hazards Ratio (HR) of Negative Outcome Controls (Atopic Dermatitis, Limb Injuries)

eFigure 1. Distribution of Test Date in SARS-CoV-2–Infected and Noninfected Patients With Cancer

eFigure 2. Distribution of Test Date in SARS-CoV-2–Infected and Noninfected Patients With Cancer (After Matching by Test Date)

eAppendix. List of *ICD-10* Codes Used for Diagnoses of Interest

This supplementary material has been provided by the authors to give readers additional information about their work.

© 2026 Wee LE et al. *JAMA Network Open*.

eTable 1. Hazards Ratio (HR) of Individual New-Incident Postacute Sequelae in SARS-CoV-2–Infected Adult Patients With Cancer and Noninfected Controls

Outcomes[c]	Proportion of SARS-CoV-2 infected cancer patients with outcome, N(%)	Proportion of test-negative cancer patients with outcome, N(%)	Hazards ratio(HR),[a] 95% CI	Excess burden(EB), weighted, per 1000-individuals,[b] 95%CI
**Post-acute diagnoses**				
**Cardiovascular diagnoses**				
Dysrhythmia	348/38342 (0.9)	279/36876 (0.8)	1.07 (0.91-1.25)	0.53 (-0.76-1.81)
Inflammatory heart disease	0/39251 (0.0)	3/37548 (0.0)	-#	-#
Ischemic heart disease	332/38264 (0.9)	313/36708 (0.9)	0.93 (0.80-1.09)	-0.60 (-1.92-0.72)
Oher cardiac disorders (eg. heart failure)[d]	76/39116 (0.2)	51/37465 (0.1)	1.22 (0.85-1.75)	0.32 (-0.25-0.89)
Thrombotic disorders	124/38901 (0.3)	120/37329 (0.3)	0.85 (0.66-1.09)	-0.53 (-1.34-0.27)
**Neurological diagnoses**				
Cerebrovascular disorders	217/38728 (0.6)	217/37127 (0.6)	0.87 (0.72-1.05)	-0.79 (-1.87-0.28)
Peripheral neuropathies	86/39033 (0.2)	79/37375 (0.2)	1.00 (0.74-1.36)	0.00 (-0.65-0.66)
Episodic disorders	74/38946 (0.2)	60/37336 (0.2)	1.06 (0.75-1.49)	0.10 (-0.49-0.68)
Movement disorders	71/38949 (0.2)	65/37317 (0.2)	0.98 (0.70-1.38)	-0.03 (-0.63-0.57)
Sensory disorders	213/38644 (0.6)	209/37044 (0.6)	0.95 (0.78-1.15)	-0.32 (-1.38-0.75)
Other neurological disorders[e]	255/38610 (0.7)	228/37072 (0.6)	0.99 (0.83-1.19)	-0.05 (-1.19-1.08)
Memory problems	187/38599 (0.5)	151/37141 (0.4)	1.13 (0.91-1.40)	0.53 (-0.42-1.48)
**Psychiatric diagnoses**				
Mood disorders	54/38923 (0.1)	50/37221 (0.1)	0.97 (0.66-1.44)	-0.04 (-0.56-0.49)
Stress and adjustment disorders	100/38910 (0.3)	89/37226 (0.2)	1.03 (0.78-1.38)	0.09 (-0.62-0.79)
Psychotic disorders	20/39110 (0.1)	14/37409 (0.0)	1.44 (0.73-2.88)	0.16 (-0.14-0.46)
**Respiratory diagnoses**				
Asthma	156/38319 (0.4)	137/36751 (0.4)	1.04 (0.82-1.31)	0.15 (-0.74-1.03)
COPD	84/38820 (0.2)	71/37123 (0.2)	0.97 (0.70-1.33)	-0.07 (-0.72-0.57)
**Gastrointestinal diagnoses**				
Appendicitis	25/39176 (0.1)	16/37465 (0.0)	1.61 (0.86-3.03)	0.26 (-0.08-0.59)
Inflammatory bowel disease	105/38989 (0.3)	88/37341 (0.2)	1.02 (0.76-1.36)	0.05 (-0.66-0.76)
Gastritis	851/35945 (2.4)	842/34649 (2.4)	0.99 (0.90-1.09)	-0.21 (-2.46-2.05)
Biliary tract disease	213/38692 (0.6)	182/37095 (0.5)	1.04 (0.85-1.27)	0.20 (-0.82-1.23)
Non-infectious hepatitis/cirrhosis	109/38965 (0.3)	71/37328 (0.2)	1.26 (0.93-1.71)	0.53 (-0.15-1.21)
**Individual post-acute symptoms**				
Cardiovascular symptoms	107/38951 (0.3)	80/37307 (0.2)	1.17 (0.87-1.57)	0.39 (-0.31-1.09)
Respiratory symptoms	362/38273 (0.9)	291/36760 (0.8)	1.09 (0.94-1.28)	0.77 (-0.55-2.09)
Headache	74/39071 (0.2)	58/37419 (0.2)	1.13 (0.80-1.60)	0.21 (-0.38-0.79)

© 2026 Wee LE et al. *JAMA Network Open*.

Musculoskeletal symptoms	488/38302 (1.3)	419/36753 (1.1)	1.07 (0.94-1.22)	0.83 (-0.74-2.39)
Loss of smell/taste	9/39233 (0.0)	6/37531 (0.0)	1.45 (0.51-4.10)	0.07 (-0.13-0.27)
Memory loss	9/39239 (0.0)	8/37545 (0.0)	0.94 (0.36-2.47)	-0.01 (-0.22-0.20)
Fatigue/malaise	0/39255 (0.0)	0/37550 (0.0)	-#	-#

HR > 1 denotes higher risk of a respective composite/individual outcome amongst COVID-19 cases and test-negatives in individuals with cancer. Excess burden > 0 denotes excess burden of a respective composite/individual outcome amongst COVID-19 cases and test-negatives in individuals with cancer.

Abbreviations: CI, confidence interval; HR, hazard ratio; EB, excess burden

[a] Competing risks regression taking death as a competing risk, with overlap weights employed. Regression adjusted based on demographic characteristics (age, sex, ethnicity), socioeconomic status (housing type), COVID-19 vaccination status, comorbidities, prior healthcare utilisation, influenza vaccination, type of cancer, time from cancer diagnosis to index date, on active cancer treatment, prevailing SARS-CoV-2 variant in circulation. Numbers in each subcohort do not add up to the original number of COVID-19 cases/test-negatives because for estimation of risks for each new-incident diagnosis, a sub-cohort of individuals without history of the diagnosis in the past 5 years was constructed.

[b] Excess-burden (EB) of new-incident sequelae per 1,000 individuals at 300 days of follow-up was defined as increase/decrease in incidence rate of new-incident sequelae amongst COVID-19 cases and test-negatives in individuals with cancer.

[c] Individual renal diagnoses were not evaluated because of the unavailability of corresponding laboratory testing results (i.e serum creatinine) to distinguish severity of renal impairment. Individual autoimmune diagnoses were not evaluated because of too few new-incident cases.

[d] Other cardiac disorders included: heart failure, non-ischemic cardiomyopathy, cardiac arrest

[e] Other neurological disorders included: dizziness, sleep disorders, somnolence, malaise and fatigue, Guillain-Barre syndrome, encephalitis/encephalopathy, transverse myelitis

-#: Could not be estimated due to too few numbers (≤1 new-incident case in any group, for that specific diagnosis)

*=p<0.05, **=p<0.01, ***=p<0.001. P-values <0.002 (individual outcomes) were taken as being additionally robust to multiple comparisons (Bonferonni)

© 2026 Wee LE et al. *JAMA Network Open*.

eTable 2. Hazards Ratio (HR) of Composite New-Incident Postacute Sequelae in Patients With Cancer Hospitalized for COVID-19, Stratified by Receipt of COVID-19 Treatment vs Noninfected Controls

Composite post-acute sequelae :	Proportion of SARS-CoV-2 infected cancer patients with outcome, N(%)	Proportion of test-negative cancer patients with outcome, N(%)	Hazards ratio(HR),[a] 95% CI	Excess burden(EB), weighted, per 1000-individuals,[b] 95%CI
**Untreated hospitalised COVID-19 cases versus all test-negatives**				
Any post-acute diagnosis[c]	138/823 (16.8)	2333/27751 (8.4)	**1.31 (1.09-1.58)*** **	36.92 (11.07-62.76)
Any cardiovascular diagnosis	73/1567 (4.7)	647/35885 (1.8)	1.30 (1.00-1.69)	10.37 (-0.13-20.87)
Any neurological diagnosis	82/1503 (5.5)	842/35347 (2.4)	**1.41 (1.10-1.80)*** **	15.17 (3.62-26.71)
Any psychiatric diagnosis	15/1800 (0.8)	134/36804 (0.4)	1.78 (1.00-3.16)	3.49 (-0.68-7.66)
Any autoimmune diagnosis	4/1849 (0.2)	44/37467 (0.1)	1.45 (0.48-4.42)	0.65 (-1.47-2.77)
Any respiratory diagnosis	19/1730 (1.1)	234/36309 (0.6)	0.91 (0.55-1.50)	-1.02 (-5.87-3.82)
Any renal diagnosis	96/1476 (6.5)	633/35169 (1.8)	**1.60 (1.26-2.03)*** **	22.82 (10.33-35.31)
Any gastrointestinal diagnosis	63/1568 (4.0)	1090/33864 (3.2)	0.99 (0.76-1.30)	-0.25 (-10.06-9.57)
Any post-acute symptom[d]	92/1543 (6.0)	987/35160 (2.8)	**1.33 (1.05-1.68)** **	14.14 (2.24-26.04)
**Treated hospitalised COVID-19 cases versus all test-negatives[f]**				
Any post-acute diagnosis[c]	135/705 (19.1)	2333/27751 (8.4)	**1.37 (1.13-1.66)*** **	45.92 (16.80-75.03)
Any cardiovascular diagnosis	99/1464 (6.8)	647/35885 (1.8)	**1.79 (1.41-2.28)*** **	29.18 (16.19-42.16)
Any neurological diagnosis	73/1353 (5.4)	842/35347 (2.4)	1.29 (0.99-1.68)	11.80 (-0.39-24.00)
Any psychiatric diagnosis	11/1659 (0.7)	134/36804 (0.4)	1.41 (0.72-2.75)	1.92 (-2.05-5.89)
Any autoimmune diagnosis	3/1700 (0.2)	44/37467 (0.1)	1.04 (0.29-3.76)	0.06 (-1.97-2.09)
Any respiratory diagnosis	22/1570 (1.4)	234/36309 (0.6)	1.25 (0.78-2.02)	2.85 (-3.09-8.79)
Any renal diagnosis	78/1317 (5.9)	633/35169 (1.8)	1.28 (0.98-1.67)	11.87 (-0.74-24.49)
Any gastrointestinal diagnosis	77/1432 (5.4)	1090/33864 (3.2)	1.37 (1.06-1.77)*	14.33 (2.40-26.26)
Any post-acute symptom[d]	105/1440 (7.3)	987/35160 (2.8)	**1.57 (1.25-1.98)*** **	25.74 (12.17-39.30)

HR > 1 denotes higher risk of a respective composite/individual outcome amongst hospitalised COVID-19 cases and test-negatives in individuals with cancer. Excess burden > 0 denotes excess burden of a respective composite/individual outcome amongst hospitalised COVID-19 cases and test-negatives in individuals with cancer.
Abbreviations: CI, confidence interval; HR, hazard ratio; EB, excess burden

[a] Competing risks regression taking death as a competing risk, with overlap weights employed. For cancer patients: overlap weighted based on demographic characteristics (age, sex, ethnicity), socioeconomic status (housing type), COVID-19 vaccination status, comorbidities, prior healthcare utilisation, influenza vaccination, type of cancer, time from cancer diagnosis to index date, on active cancer treatment, prevailing SARS-CoV-2 variant in circulation. Numbers in each subcohort do not add up to the original number of COVID-19 cases/test-negatives because for estimation of risks for each new-incident diagnosis, a sub-cohort of individuals without history of the diagnosis in the past 5 years was constructed.

© 2026 Wee LE et al. *JAMA Network Open*.

[b] Excess-burden (EB) of new-incident sequelae per 1,000 individuals at 300 days of follow-up was defined as increase/decrease in incidence rate of new-incident sequelae amongst COVID-19 cases and test-negatives in individuals with cancer.
[c] Any post-acute diagnosis was taken as a composite of any cardiovascular/neurological/psychiatric/autoimmune/respiratory/renal/gastrointestinal diagnoses in cancer patients.
[d] Any post-acute symptom was taken as a composite of any individual post-acute symptoms, including cardiovascular signs/symptoms, respiratory signs/symptoms, headache, musculoskeletal pain/stiffness, abdominal/pelvic pain, generalised pain, loss of smell or taste, memory and cognitive impairment, fatigue and malaise.
[e] COVID-19 hospitalisation was defined as any hospitalisation attributed to COVID-19, reported in the national COVID-19 database.
[f] COVID-19 treatment was defined as receipt of any COVID-19 antiviral (remdesivir, molnupiravir, nirmatrelvir-ritonavir) or monoclonal antibody (sotrovimab, tixagevimab/cilgavimab) administered within 7 days of T_0, index-date; receipt of COVID-19 therapeutics was tracked using data available to the local Ministry of Health.
*=p<0.05, **=p<0.01, ***=p<0.001. P-values<0.005 (composite outcomes) were taken as being additionally robust to multiple comparisons (Bonferonni)

© 2026 Wee LE et al. *JAMA Network Open*.

eTable 3. Hazards Ratio (HR) of Composite New-Incident Postacute Sequelae in SARS-CoV-2–Infected Patients With Cancer and Noninfected Controls, Using Alternative Weighting Scheme (Inverse Propensity Weighting)

**Composite post-acute sequelae :**	**Proportion of SARS-CoV-2 infected cancer patients with outcome, N(%)**	**Proportion of test-negative cancer patients with outcome, N(%)**	**Hazards ratio(HR),[a] 95% CI**	**Excess burden(EB), weighted, per 1000-individuals,[b] 95%CI**
**Cancer patients with COVID-19, all**				
Any post-acute diagnosis[c]	2320/27635 (8.4)	2333/27751 (8.4)	0.98 (0.92-1.04)	-1.90 (-6.53-2.73)
Any cardiovascular diagnosis	732/37096 (2.0)	647/35885 (1.8)	0.99 (0.89-1.10)	-0.16 (-2.14-1.82)
Any neurological diagnosis	905/36331 (2.5)	842/35347 (2.4)	1.00 (0.91-1.10)	-0.08 (-2.34-2.19)
Any psychiatric diagnosis	157/38483 (0.4)	134/36804 (0.4)	1.09 (0.86-1.37)	0.32 (-0.57-1.21)
Any autoimmune diagnosis	54/39151 (0.1)	44/37467 (0.1)	1.16 (0.77-1.73)	0.19 (-0.32-0.69)
Any respiratory diagnosis	269/37871 (0.7)	234/36309 (0.6)	1.02 (0.85-1.21)	0.11 (-1.07-1.29)
Any renal diagnosis	732/36044 (2.0)	633/35169 (1.8)	1.02 (0.91-1.13)	0.30 (-1.72-2.31)
Any gastrointestinal diagnosis	1161/34988 (3.3)	1090/33864 (3.2)	1.02 (0.94-1.11)	0.80 (-1.85-3.46)
Any post-acute symptom[d]	1177/36317 (3.2)	987/35160 (2.8)	1.09 (1.00-1.19)	2.65 (0.14-5.17)
**Cancer patients hospitalised for COVID-19[e]**				
Any post-acute diagnosis[c]	273/1528 (17.9)	2333/27751 (8.4)	**1.84 (1.45-2.34)*** **	67.96 (49.49-86.42)
Any cardiovascular diagnosis	172/3031 (5.7)	647/35885 (1.8)	**2.27 (1.61-3.22)*** **	24.82 (17.32-32.31)
Any neurological diagnosis	155/2856 (5.4)	842/35347 (2.4)	1.58 (1.21-2.06)	14.25 (6.93-21.57)
Any psychiatric diagnosis	26/3459 (0.8)	134/36804 (0.4)	1.48 (0.61-3.60)	1.78 (-0.76-4.32)
Any autoimmune diagnosis	7/3549 (0.2)	44/37467 (0.1)	1.73 (0.52-5.78)	0.89 (-0.66-2.43)
Any respiratory diagnosis	41/3300 (1.2)	234/36309 (0.6)	**2.13 (1.09-4.18)***	7.71 (3.53-11.89)
Any renal diagnosis	174/2793 (6.2)	633/35169 (1.8)	**1.92 (1.47-2.52)*** **	18.63 (11.28-25.98)
Any gastrointestinal diagnosis	140/3000 (4.7)	1090/33864 (3.2)	1.18 (0.89-1.57)	5.93 (-1.24-13.11)
Any post-acute symptom[d]	197/2983 (6.6)	987/35160 (2.8)	**1.88 (1.48-2.39)*** **	25.49 (17.10-33.89)
**Cancer patients with COVID-19 not requiring initial hospitalisation**				
Any post-acute diagnosis[c]	2047/26107 (7.8)	2333/27751 (8.4)	0.95 (0.89-1.00)	-4.37 (-9.00-0.26)
Any cardiovascular diagnosis	560/34065 (1.6)	647/35885 (1.8)	0.91 (0.81-1.02)	-1.70 (-3.63-0.24)
Any neurological diagnosis	750/33475 (2.2)	842/35347 (2.4)	0.95 (0.86-1.05)	-1.05 (-3.31-1.20)
Any psychiatric diagnosis	131/35024 (0.4)	134/36804 (0.4)	1.04 (0.82-1.33)	0.16 (-0.73-1.06)
Any autoimmune diagnosis	47/35602 (0.1)	44/37467 (0.1)	1.14 (0.75-1.72)	0.16 (-0.35-0.68)
Any respiratory diagnosis	228/34571 (0.7)	234/36309 (0.6)	1.00 (0.84-1.21)	0.03 (-1.16-1.21)
Any renal diagnosis	558/33251 (1.7)	633/35169 (1.8)	0.93 (0.83-1.04)	-1.30 (-3.26-0.67)

© 2026 Wee LE et al. *JAMA Network Open*.

Any gastrointestinal diagnosis	1021/31988 (3.2)	1090/33864 (3.2)	1.01 (0.93-1.10)	0.29 (-2.41-2.98)
Any post-acute symptom[d]	980/33334 (2.9)	987/35160 (2.8)	1.05 (0.96-1.15)	1.34 (-1.16-3.85)

HR > 1 denotes higher risk of a respective composite/individual outcome amongst COVID-19 cases and test-negatives in individuals with cancer. Excess burden > 0 denotes excess burden of a respective composite/individual outcome amongst COVID-19 cases and test-negatives in individuals with cancer.
Abbreviations: CI, confidence interval; HR, hazard ratio; EB, excess burden

[a] Competing risks regression taking death as a competing risk, with inverse-propensity-treatment-weighting (IPTW) employed. For cancer patients: weighted based on demographic characteristics (age, sex, ethnicity), socioeconomic status (housing type), COVID-19 vaccination status, comorbidities, prior healthcare utilisation, prior influenza vaccination, type of cancer, time from cancer diagnosis to index date, on active cancer treatment, prevailing SARS-CoV-2 variant in circulation. Numbers in each subcohort do not add up to the original number of COVID-19 cases/test-negatives because for estimation of risks for each new-incident diagnosis, a sub-cohort of individuals without history of the diagnosis in the past 5 years was constructed.
[b] Excess-burden (EB) of new-incident sequelae per 1,000 individuals at 300 days of follow-up was defined as increase/decrease in incidence rate of new-incident sequelae amongst COVID-19 cases and test-negatives in individuals with cancer.
[c] Any post-acute diagnosis was taken as a composite of any cardiovascular/neurological/psychiatric/autoimmune/respiratory/renal/gastrointestinal diagnoses in cancer patients.
[d] Any post-acute symptom was taken as a composite of any individual post-acute symptoms, including cardiovascular signs/symptoms, respiratory signs/symptoms, headache, musculoskeletal pain/stiffness, abdominal/pelvic pain, generalised pain, loss of smell or taste, memory and cognitive impairment, fatigue and malaise.
[e] COVID-19 hospitalisation was defined as any hospitalisation attributed to COVID-19, reported in the national COVID-19 database.
*=p<0.05, **=p<0.01, ***=p<0.001. P-values<0.005 (composite outcomes) and p-values<0.002 (individual outcomes) were taken as being additionally robust to multiple comparisons (Bonferonni)
-#: Could not be estimated due to too few numbers (≤1 new-incident case in any group, for that specific diagnosis)

© 2026 Wee LE et al. *JAMA Network Open*.

eTable 4. Adjusted Hazards Ratio (AHR) of Composite New-Incident Postacute Sequelae in SARS-CoV-2–Infected Patients With Cancer and Noninfected Controls Using Competing Risks Regression (Unweighted)

**Composite post-acute sequelae :**	**Proportion of SARS-CoV-2 infected cancer patients with outcome, N(%)**	**Proportion of test-negative cancer patients with outcome, N(%)**	**Adjusted-hazards ratio(aHR),[a] 95% CI**	**Excess burden(EB), per 1000-individuals,[b] 95%CI**
**Cancer patients with COVID-19, all**				
Any post-acute diagnosis[c]	2320/27635 (8.4)	2333/27751 (8.4)	1.00 (0.94-1.06)	-0.12 (-4.74-4.50)
Any cardiovascular diagnosis	732/37096 (2.0)	647/35885 (1.8)	1.10 (0.99-1.22)	1.70 (-0.27-3.68)
Any neurological diagnosis	905/36331 (2.5)	842/35347 (2.4)	1.05 (0.95-1.15)	1.09 (-1.17-3.35)
Any psychiatric diagnosis	157/38483 (0.4)	134/36804 (0.4)	1.12 (0.89-1.41)	0.44 (-0.45-1.32)
Any autoimmune diagnosis	54/39151 (0.1)	44/37467 (0.1)	1.17 (0.79-1.75)	0.20 (-0.30-0.71)
Any respiratory diagnosis	269/37871 (0.7)	234/36309 (0.6)	1.10 (0.93-1.31)	0.66 (-0.52-1.84)
Any renal diagnosis	732/36044 (2.0)	633/35169 (1.8)	**1.13 (1.02-1.26)***	2.31 (0.30-4.32)
Any gastrointestinal diagnosis	1161/34988 (3.3)	1090/33864 (3.2)	1.03 (0.95-1.12)	1.00 (-1.66-3.65)
Any post-acute symptom[d]	1177/36317 (3.2)	987/35160 (2.8)	**1.16 (1.06-1.26)****	4.34 (1.83-6.85)
**Cancer patients hospitalised for COVID-19[e]**				
Any post-acute diagnosis[c]	273/1528 (17.9)	2333/27751 (8.4)	**2.26 (1.99-2.56)*****	94.60 (75.11-114.08)
Any cardiovascular diagnosis	172/3031 (5.7)	647/35885 (1.8)	**3.22 (2.72-3.81)*****	38.72 (30.37-47.07)
Any neurological diagnosis	155/2856 (5.4)	842/35347 (2.4)	**2.32 (1.95-2.75)*****	30.45 (21.99-38.91)
Any psychiatric diagnosis	26/3459 (0.8)	134/36804 (0.4)	**2.07 (1.36-3.15)*****	3.88 (0.93-6.82)
Any autoimmune diagnosis	7/3549 (0.2)	44/37467 (0.1)	1.68 (0.76-3.73)	0.80 (-0.70-2.30)
Any respiratory diagnosis	41/3300 (1.2)	234/36309 (0.6)	**1.93 (1.39-2.70)*****	5.98 (2.11-9.85)
Any renal diagnosis	174/2793 (6.2)	633/35169 (1.8)	**3.55 (3.00-4.19)*****	44.30 (35.23-53.37)
Any gastrointestinal diagnosis	140/3000 (4.7)	1090/33864 (3.2)	**1.46 (1.23-1.74)*****	14.48 (6.70-22.26)
Any post-acute symptom[d]	197/2983 (6.6)	987/35160 (2.8)	**2.40 (2.06-2.80)*****	37.97 (28.89-47.05)
**Cancer patients with COVID-19 not requiring initial hospitalisation**				
Any post-acute diagnosis[c]	2047/26107 (7.8)	2333/27751 (8.4)	0.93 (0.88-1.01)	-5.66 (-10.28--1.05)
Any cardiovascular diagnosis	560/34065 (1.6)	647/35885 (1.8)	0.91 (0.81-1.02)	-1.59 (-3.52-0.34)
Any neurological diagnosis	750/33475 (2.2)	842/35347 (2.4)	0.94 (0.85-1.04)	-1.42 (-3.66-0.83)
Any psychiatric diagnosis	131/35024 (0.4)	134/36804 (0.4)	1.03 (0.81-1.31)	0.10 (-0.79-0.99)
Any autoimmune diagnosis	47/35602 (0.1)	44/37467 (0.1)	1.12 (0.75-1.70)	0.15 (-0.37-0.66)
Any respiratory diagnosis	228/34571 (0.7)	234/36309 (0.6)	1.02 (0.85-1.23)	0.15 (-1.04-1.34)
Any renal diagnosis	558/33251 (1.7)	633/35169 (1.8)	0.93 (0.83-1.04)	-1.22 (-3.18-0.74)
Any gastrointestinal diagnosis	1021/31988 (3.2)	1090/33864 (3.2)	0.99 (0.91-1.08)	-0.27 (-2.96-2.42)

© 2026 Wee LE et al. *JAMA Network Open*.

Any post-acute symptom[d]	980/33334 (2.9)	987/35160 (2.8)	1.05 (0.96-1.14)	1.33 (-1.18-3.83)

HR > 1 denotes higher risk of a respective composite/individual outcome amongst COVID-19 cases and test-negatives in individuals with cancer. Excess burden > 0 denotes excess burden of a respective composite/individual outcome amongst COVID-19 cases and test-negatives in individuals with cancer.
Abbreviations: CI, confidence interval; HR, hazard ratio; EB, excess burden

[a] Competing risks regression taking death as a competing risk; regression adjusted for the following covariates: demographic characteristics (age, sex, ethnicity), socioeconomic status (housing type), COVID-19 vaccination status, comorbidities, prior healthcare utilisation, prior influenza vaccination, type of cancer, time from cancer diagnosis to index date, on active cancer treatment, prevailing SARS-CoV-2 variant in circulation. Numbers in each subcohort do not add up to the original number of COVID-19 cases/test-negatives because for estimation of risks for each new-incident diagnosis, a sub-cohort of individuals without history of the diagnosis in the past 5 years was constructed.
[b] Excess-burden (EB) of new-incident sequelae per 1,000 individuals at 300 days of follow-up was defined as increase/decrease in incidence rate of new-incident sequelae amongst COVID-19 cases and test-negatives in individuals with cancer.
[c] Any post-acute diagnosis was taken as a composite of any cardiovascular/neurological/psychiatric/autoimmune/respiratory/renal/gastrointestinal diagnoses in cancer patients.
[d] Any post-acute symptom was taken as a composite of any individual post-acute symptoms, including cardiovascular signs/symptoms, respiratory signs/symptoms, headache, musculoskeletal pain/stiffness, abdominal/pelvic pain, generalised pain, loss of smell or taste, memory and cognitive impairment, fatigue and malaise.
[e] COVID-19 hospitalisation was defined as any hospitalisation attributed to COVID-19, reported in the national COVID-19 database.
*=p<0.05, **=p<0.01, ***=p<0.001. P-values<0.005 (composite outcomes) and p-values<0.002 (individual outcomes) were taken as being additionally robust to multiple comparisons (Bonferonni)
-#: Could not be estimated due to too few numbers (≤1 new-incident case in any group, for that specific diagnosis)

© 2026 Wee LE et al. *JAMA Network Open*.

eTable 5. Adjusted Hazards Ratio (AHR) of Composite New-Incident Postacute Sequelae in SARS-CoV-2–Infected Patients With Cancer and Noninfected Controls After Cessation of Routine Rostered Testing for Asymptomatic Individuals

Composite post-acute sequelae :	Proportion of SARS-CoV-2 infected cancer patients with outcome, N(%)	Proportion of test-negative cancer patients with outcome, N(%)	Hazards ratio(HR),[a] 95% CI	Excess burden(EB), weighted, per 1000-individuals,[b] 95%CI
**Cancer patients with COVID-19, all**				
Any post-acute diagnosis[c]	1337/15803 (8.5)	1260/15525 (8.1)	1.04 (0.97-1.13)	3.37 (-2.74-9.47)
Any cardiovascular diagnosis	406/21192 (1.9)	369/20175 (1.8)	0.98 (0.85-1.13)	-0.32 (-2.92-2.28)
Any neurological diagnosis	542/20719 (2.6)	468/19881 (2.4)	1.08 (0.95-1.23)	1.93 (-1.09-4.96)
Any psychiatric diagnosis	86/21954 (0.4)	73/20739 (0.4)	1.04 (0.76-1.42)	0.14 (-1.03-1.30)
Any autoimmune diagnosis	34/22326 (0.2)	32/21097 (0.2)	1.05 (0.65-1.72)	0.08 (-0.67-0.83)
Any respiratory diagnosis	154/21624 (0.7)	130/20455 (0.6)	1.05 (0.83-1.33)	0.35 (-1.21-1.91)
Any renal diagnosis	424/20663 (2.1)	353/19700 (1.8)	1.06 (0.92-1.22)	1.11 (-1.55-3.78)
Any gastrointestinal diagnosis	676/19854 (3.4)	582/19098 (3.0)	1.13 (1.00-1.26)	3.80 (0.29-7.30)
Any post-acute symptom[d]	692/20652 (3.4)	585/19778 (3.0)	1.09 (0.97-1.22)	2.59 (-0.81-5.99)
**Cancer patients hospitalised for COVID-19[e]**				
Any post-acute diagnosis[c]	166/881 (18.8)	1260/15525 (8.1)	**1.48 (1.23-1.79)*****	54.89 (28.89-80.89)
Any cardiovascular diagnosis	104/1778 (5.8)	369/20175 (1.8)	**1.59 (1.23-2.05)*****	20.57 (9.54-31.61)
Any neurological diagnosis	104/1659 (6.3)	468/19881 (2.4)	**1.53 (1.20-1.95)****	20.32 (8.57-32.07)
Any psychiatric diagnosis	19/2036 (0.9)	73/20739 (0.4)	1.69 (0.95-2.99)	3.38 (-0.68-7.43)
Any autoimmune diagnosis	3/2086 (0.1)	32/21097 (0.2)	0.70 (0.19-2.54)	-0.55 (-2.21-1.10)
Any respiratory diagnosis	24/1919 (1.3)	130/20455 (0.6)	1.07 (0.66-1.75)	0.77 (-4.16-5.69)
Any renal diagnosis	116/1683 (6.9)	353/19700 (1.8)	**1.62 (1.26-2.07)*****	24.14 (12.06-36.21)
Any gastrointestinal diagnosis	79/1764 (4.5)	582/19098 (3.0)	1.27 (0.97-1.66)	9.15 (-0.77-19.06)
Any post-acute symptom[d]	118/1746 (6.8)	585/19778 (3.0)	**1.45 (1.16-1.83)*****	20.43 (8.36-32.50)
**Cancer patients with COVID-19 not requiring initial hospitalisation**				
Any post-acute diagnosis[c]	1171/14922 (7.8)	1260/15525 (8.1)	1.01 (0.93-1.09)	0.41 (-5.68-6.51)
Any cardiovascular diagnosis	302/19414 (1.6)	369/20175 (1.8)	0.88 (0.76-1.03)	-2.11 (-4.64-0.42)
Any neurological diagnosis	438/19060 (2.3)	468/19881 (2.4)	1.02 (0.90-1.16)	0.48 (-2.51-3.48)
Any psychiatric diagnosis	67/19918 (0.3)	73/20739 (0.4)	0.95 (0.68-1.33)	-0.17 (-1.32-0.99)
Any autoimmune diagnosis	31/20240 (0.2)	32/21097 (0.2)	1.08 (0.65-1.77)	0.12 (-0.65-0.88)
Any respiratory diagnosis	130/19705 (0.7)	130/20455 (0.6)	1.04 (0.82-1.34)	0.28 (-1.29-1.85)
Any renal diagnosis	308/18980 (1.6)	353/19700 (1.8)	0.94 (0.81-1.10)	-0.96 (-3.54-1.62)

© 2026 Wee LE et al. *JAMA Network Open*.

Any gastrointestinal diagnosis	597/18090 (3.3)	582/19098 (3.0)	1.11 (0.99-1.25)	3.42 (-0.15-7.00)
Any post-acute symptom[d]	574/18906 (3.0)	585/19778 (3.0)	1.05 (0.93-1.17)	1.31 (-2.09-4.72)

1st April-31st December 2022
HR > 1 denotes higher risk of a respective composite/individual outcome amongst COVID-19 cases and test-negatives in individuals with cancer. Excess burden > 0 denotes excess burden of a respective composite/individual outcome amongst COVID-19 cases and test-negatives in individuals with cancer. *=p<0.05, **=p<0.01, ***=p<0.001. P-values<0.005 (composite outcomes) and p-values<0.002 (individual outcomes) were taken as being additionally robust to multiple comparisons (Bonferonni). -# denotes that HRs could not be estimated due to too few numbers (≤1 new-incident case in any group, for that specific diagnosis)

Abbreviations: CI, confidence interval; HR, hazard ratio; EB, excess burden

[a] Competing risks regression taking death as a competing risk, with overlap weights employed. For cancer patients: overlap weighted based on demographic characteristics (age, sex, ethnicity), socioeconomic status (housing type), COVID-19 vaccination status, comorbidities, prior healthcare utilisation, prior influenza vaccination, type of cancer, time from cancer diagnosis to index date, on active cancer treatment, prevailing SARS-CoV-2 variant in circulation. Numbers in each subcohort do not add up to the original number of COVID-19 cases/test-negatives because for estimation of risks for each new-incident diagnosis, a sub-cohort of individuals without history of the diagnosis in the past 5 years was constructed.
[b] Excess-burden (EB) of new-incident sequelae per 1,000 individuals at 300 days of follow-up was defined as increase/decrease in incidence rate of new-incident sequelae amongst COVID-19 cases and test-negatives in individuals with cancer.
[c] Any post-acute diagnosis was taken as a composite of any cardiovascular/neurological/psychiatric/autoimmune/respiratory/renal/gastrointestinal diagnoses in cancer patients.
[d] Any post-acute symptom was taken as a composite of any individual post-acute symptoms, including cardiovascular signs/symptoms, respiratory signs/symptoms, headache, musculoskeletal pain/stiffness, abdominal/pelvic pain, generalised pain, loss of smell or taste, memory and cognitive impairment, fatigue and malaise.
[e] COVID-19 hospitalisation was defined as any hospitalisation attributed to COVID-19, reported in the national COVID-19 database.

© 2026 Wee LE et al. *JAMA Network Open*.

eTable 6. Adjusted Hazards Ratio (AHR) of Composite New-Incident Postacute Sequelae in SARS-CoV-2–Infected Patients With Cancer and Noninfected Controls in Subset of Solid Organ Cancer Cases With Staging Data Available

Composite post-acute sequelae :	Proportion of SARS-CoV-2 infected cancer patients with outcome, N(%)	Proportion of test-negative cancer patients with outcome, N(%)	Hazards ratio(HR),[a] 95% CI	Excess burden(EB), weighted, per 1000-individuals,[b] 95%CI
**Cancer patients with COVID-19, all**				
Any post-acute diagnosis[c]	1237/16564 (7.5)	1159/16356 (7.1)	1.06 (0.98-1.15)	4.00 (-1.70-9.71)
Any cardiovascular diagnosis	343/20988 (1.6)	282/19934 (1.4)	1.18 (1.00-1.38)	2.47 (0.00-4.94)
Any neurological diagnosis	355/20905 (1.7)	355/19910 (1.8)	0.96 (0.83-1.12)	-0.71 (-3.34-1.92)
Any psychiatric diagnosis	30/21972 (0.1)	17/20630 (0.1)	1.69 (0.93-3.07)	0.56 (0.08-1.20)
Any autoimmune diagnosis	11/22020 (0.0)	8/20681 (0.0)	1.26 (0.50-3.16)	0.11 (-0.32-0.54)
Any respiratory diagnosis	75/21877 (0.3)	53/20529 (0.3)	1.36 (0.96-1.94)	0.93 (-0.15-2.01)
Any renal diagnosis	262/21272 (1.2)	196/20193 (1.0)	**1.29 (1.07-1.56)***	2.89 (0.75-5.03)
Any gastrointestinal diagnosis	608/19727 (3.1)	528/18722 (2.8)	1.10 (0.98-1.24)	2.73 (-0.70-6.15)
Any post-acute symptom[d]	475/20613 (2.3)	344/19614 (1.8)	**1.32 (1.15-1.52)*****	5.72 (2.85-8.59)
**Cancer patients hospitalised for COVID-19[e]**				
Any post-acute diagnosis[c]	182/1044 (17.4)	1159/16356 (7.1)	**1.91 (1.54-2.36)*****	73.50 (41.26-105.75)
Any cardiovascular diagnosis	101/1817 (5.6)	282/19934 (1.4)	**2.46 (1.84-3.28)*****	34.09 (19.45-48.73)
Any neurological diagnosis	62/1830 (3.4)	355/19910 (1.8)	1.36 (0.98-1.90)	7.65 (-3.75-19.06)
Any psychiatric diagnosis	5/2091 (0.2)	17/20630 (0.1)	1.67 (0.46-6.15)	0.60 (-1.31-2.50)
Any autoimmune diagnosis	3/2107 (0.1)	8/20681 (0.0)	1.59 (0.29-8.63)	0.43 (-1.50-2.36)
Any respiratory diagnosis	19/2053 (0.9)	53/20529 (0.3)	**2.55 (1.33-4.88)*****	6.61 (0.39-12.84)
Any renal diagnosis	90/1818 (5.0)	196/20193 (1.0)	**2.87 (2.11-3.90)*****	36.73 (22.13-51.33)
Any gastrointestinal diagnosis	79/1797 (4.4)	528/18722 (2.8)	**1.54 (1.14-2.09)*****	13.78 (1.16-26.40)
Any post-acute symptom[d]	108/1782 (6.1)	344/19614 (1.8)	**2.73 (2.07-3.60)*****	41.49 (25.76-57.21)
**Cancer patients with COVID-19 not requiring initial hospitalisation**				
Any post-acute diagnosis[c]	1055/15520(6.8)	1159/16356 (7.1)	0.95 (0.88-1.04)	-2.98 (-8.56-2.60)
Any cardiovascular diagnosis	242/19171 (1.3)	282/19934 (1.4)	0.90(0.76-1.07)	-1.40 (-3.67-0.86)
Any neurological diagnosis	293/19075 (1.5)	355/19910 (1.8)	0.86 (0.74-1.01)	-2.39 (-4.93-0.15)
Any psychiatric diagnosis	25/19881 (0.1)	17/20630 (0.1)	1.54 (0.83-2.87)	0.44 (-0.19-1.08)
Any autoimmune diagnosis	8/19913 (0.0)	8/20681 (0.0)	1.01 (0.38-2.70)	0.00 (-0.40-0.40)
Any respiratory diagnosis	56/19824 (0.3)	53/20529 (0.3)	1.10 (0.75-1.60)	0.24 (-0.75-1.23)

© 2026 Wee LE et al. *JAMA Network Open*.

Any renal diagnosis	172/19454 (0.9)	196/20193 (1.0)	0.92 (0.75-1.13)	-0.75 (-2.66-1.16)
Any gastrointestinal diagnosis	529/17930 (3.0)	528/18722(2.8)	1.04 (0.92-1.18)	1.24 (-2.20-4.69)
Any post-acute symptom[d]	367/18831 (1.9)	344/19614 (1.8)	1.09 (0.94-1.27)	1.69 (-1.03-.41)

HR > 1 denotes higher risk of a respective composite/individual outcome amongst COVID-19 cases and test-negatives in individuals with cancer. Excess burden > 0 denotes excess burden of a respective composite/individual outcome amongst COVID-19 cases and test-negatives in individuals with cancer. *=p<0.05, **=p<0.01, ***=p<0.001. P-values<0.005 (composite outcomes) and p-values<0.002 (individual outcomes) were taken as being additionally robust to multiple comparisons (Bonferonni). -# denotes that HRs could not be estimated due to too few numbers (⩽1 new-incident case in any group, for that specific diagnosis)

Abbreviations: CI, confidence interval; HR, hazard ratio; EB, excess burden

[a] Competing risks regression taking death as a competing risk, with overlap weights employed. For cancer patients: overlap weighted based on demographic characteristics (age, sex, ethnicity), socioeconomic status (housing type), COVID-19 vaccination status, comorbidities, prior healthcare utilisation, prior influenza vaccination, type of cancer, cancer stage (Stage I/II: locally extensive spread; Stage III: regional nodal spread; Stage IV: distant metastasis); time from cancer diagnosis to index date, on active cancer treatment, prevailing SARS-CoV-2 variant in circulation. Numbers in each subcohort do not add up to the original number of COVID-19 cases/test-negatives because for estimation of risks for each new-incident diagnosis, a sub-cohort of individuals without history of the diagnosis in the past 5 years was constructed. Numbers differ from the original cohort because individuals with missing information on staging were excluded from this sensitivity analysis. Stage I/II: locally extensive spread; Stage III: regional nodal spread; Stage IV: distant metastasis.
[b] Excess-burden (EB) of new-incident sequelae per 1,000 individuals at 300 days of follow-up was defined as increase/decrease in incidence rate of new-incident sequelae amongst COVID-19 cases and test-negatives in individuals with cancer.
[c] Any post-acute diagnosis was taken as a composite of any cardiovascular/neurological/psychiatric/autoimmune/respiratory/renal/gastrointestinal diagnoses in cancer patients.
[d] Any post-acute symptom was taken as a composite of any individual post-acute symptoms, including cardiovascular signs/symptoms, respiratory signs/symptoms, headache, musculoskeletal pain/stiffness, abdominal/pelvic pain, generalised pain, loss of smell or taste, memory and cognitive impairment, fatigue and malaise.
[e] COVID-19 hospitalisation was defined as any hospitalisation attributed to COVID-19, reported in the national COVID-19 database.

© 2026 Wee LE et al. *JAMA Network Open*.

eTable 7. Adjusted Hazards Ratio (AHR) of Composite New-Incident Postacute Sequelae in SARS-CoV-2–Infected Patients With Cancer and Noninfected Controls Matched by Time 0

Composite post-acute sequelae :	Proportion of SARS-CoV-2 infected cancer patients with outcome, N(%)	Proportion of test-negative cancer patients with outcome, N(%)	Hazards ratio(HR),[a] 95% CI	Excess burden(EB), weighted, per 1000-individuals,[b] 95%CI
**Cancer patients with COVID-19, all**				
Any post-acute diagnosis[c]	2320/27635 (8.4)	1015/11991 (8.5)	0.97 (0.90-1.05)	-2.12 (-8.08-3.85)
Any cardiovascular diagnosis	732/37096 (2.0)	280/15445 (1.8)	0.99 (0.86-1.14)	-0.20 (-2.73-2.33)
Any neurological diagnosis	905/36331 (2.5)	376/15241 (2.5)	0.96 (0.85-1.09)	-0.87 (-3.81-2.06)
Any psychiatric diagnosis	157/38483 (0.4)	47/15850 (0.3)	1.34 (0.96-1.86)	1.01 (-0.05-2.07)
Any autoimmune diagnosis	54/39061 (0.1)	26/16136 (0.2)	0.84 (0.52-1.35)	-0.26 (-0.98-0.46)
Any respiratory diagnosis	269/37871 (0.7)	92/15662 (0.6)	1.10 (0.87-1.40)	0.62 (-0.85-2.09)
Any renal diagnosis	732/36044 (2.0)	278/15124 (1.8)	0.99 (0.86-1.13)	-0.27 (-2.86-2.31)
Any gastrointestinal diagnosis	1161/34988 (3.3)	460/14631 (3.1)	1.05 (0.94-1.17)	1.44 (-1.95-4.83)
Any post-acute symptom[d]	1177/36317 (3.2)	418/15138 (2.8)	1.12 (1.00-1.25)	3.17 (0.00-6.34)
**Hospitalised for COVID-19[e]**				
Any post-acute diagnosis[c]	273/1528 (17.9)	1015/11991 (8.5)	**1.42 (1.21-1.66)*** **	46.79 (26.95-66.62)
Any cardiovascular diagnosis	172/3031 (5.7)	280/15445 (1.8)	**1.68 (1.34-2.11)*** **	20.71 (12.35-29.08)
Any neurological diagnosis	155/2856 (5.4)	376/15241 (2.5)	**1.45 (1.16-1.81)*** **	15.59 (6.99-24.19)
Any psychiatric diagnosis	26/3459 (0.8)	47/15850 (0.3)	**2.02 (1.10-3.71)* **	3.32 (0.48-6.16)
Any autoimmune diagnosis	7/3549 (0.2)	26/16136 (0.2)	1.00 (0.38-2.64)	0.01 (-1.58-1.59)
Any respiratory diagnosis	41/3300 (1.2)	92/15662 (0.6)	1.11 (0.72-1.72)	1.17 (-2.82-5.16)
Any renal diagnosis	174/2793 (6.2)	278/15124 (1.8)	**1.50 (1.20-1.88)*** **	17.84 (8.89-26.79)
Any gastrointestinal diagnosis	140/3000 (4.7)	460/14631 (3.1)	1.16 (0.92-1.45)	5.91 (-2.13-13.94)
Any post-acute symptom[d]	197/2983 (6.6)	418/15138 (2.8)	**1.64 (1.33-2.02)*** **	24.24 (14.97-33.51)
**Not requiring initial hospitalisation**				
Any post-acute diagnosis[c]	2047/26107 (7.8)	1015/11991 (8.5)	0.94 (0.87-1.02)	-4.73 (-10.70-1.23)
Any cardiovascular diagnosis	560/34065 (1.6)	280/15445 (1.8)	0.90 (0.78-1.04)	-1.74 (-4.25-0.76)
Any neurological diagnosis	750/33475 (2.2)	376/15241 (2.5)	0.92 (0.81-1.04)	-1.87 (-4.81-1.06)
Any psychiatric diagnosis	131/35024 (0.4)	47/15850 (0.3)	1.29 (0.92-1.80)	0.86 (-0.21-1.92)
Any autoimmune diagnosis	47/35602 (0.1)	26/16136 (0.2)	0.82 (0.51-1.34)	-0.28 (-1.01-0.44)
Any respiratory diagnosis	228/34571 (0.7)	92/15662 (0.6)	1.09 (0.85-1.38)	0.51 (-0.96-1.99)
Any renal diagnosis	558/33251 (1.7)	278/15124 (1.8)	0.90 (0.78-1.04)	-1.80 (-4.35-0.75)
Any gastrointestinal diagnosis	1021/31988 (3.2)	460/14631 (3.1)	1.03 (0.92-1.15)	0.99 (-2.43-4.42)
Any post-acute symptom[d]	980/33334 (2.9)	418/15138 (2.8)	1.07 (0.95-1.20)	1.87 (-1.31-5.04)

© 2026 Wee LE et al. *JAMA Network Open*.

HR > 1 denotes higher risk of a respective composite/individual outcome amongst COVID-19 cases and test-negatives in individuals with cancer, with test-negatives matched by T0. Excess burden > 0 denotes excess burden of a respective composite/individual outcome amongst COVID-19 cases and test-negatives in individuals with cancer. *=p<0.05, **=p<0.01, ***=p<0.001. P-values<0.005 (composite outcomes) and p-values<0.002 (individual outcomes) were taken as being additionally robust to multiple comparisons (Bonferonni). -# denotes that HRs could not be estimated due to too few numbers (⩽1 new-incident case in any group, for that specific diagnosis)

Abbreviations: CI, confidence interval; HR, hazard ratio; EB, excess burden

[a] Competing risks regression taking death as a competing risk, with overlap weights employed. For cancer patients: overlap weighted based on demographic characteristics (age, sex, ethnicity), socioeconomic status (housing type), COVID-19 vaccination status, comorbidities, prior healthcare utilisation, prior influenza vaccination, type of cancer, time from cancer diagnosis to index date, on active cancer treatment, prevailing SARS-CoV-2 variant in circulation. Numbers in each subcohort do not add up to the original number of COVID-19 cases/test-negatives because for estimation of risks for each new-incident diagnosis, a sub-cohort of individuals without history of the diagnosis in the past 5 years was constructed.
[b] Excess-burden (EB) of new-incident sequelae per 1,000 individuals at 300 days of follow-up was defined as increase/decrease in incidence rate of new-incident sequelae amongst COVID-19 cases and test-negatives in individuals with cancer.
[c] Any post-acute diagnosis was taken as a composite of any cardiovascular/neurological/psychiatric/autoimmune/respiratory/renal/gastrointestinal diagnoses in cancer patients.
[d] Any post-acute symptom was taken as a composite of any individual post-acute symptoms, including cardiovascular signs/symptoms, respiratory signs/symptoms, headache, musculoskeletal pain/stiffness, abdominal/pelvic pain, generalised pain, loss of smell or taste, memory and cognitive impairment, fatigue and malaise.
[e] COVID-19 hospitalisation was defined as any hospitalisation attributed to COVID-19, reported in the national COVID-19 database.

© 2026 Wee LE et al. *JAMA Network Open*.

eTable 8. Hazards Ratio (HR) of Composite New-Incident Postacute Sequelae in Patients With Cancer Hospitalized for COVID-19 vs Influenza Hospitalizations, Noninfected Controls Hospitalized for Respiratory Complications, and Noninfected Controls Hospitalized for Any Cause

**Composite post-acute sequelae :**	**Proportion of cancer patients hospitalised for COVID-19[a] with outcome, N(%)**	**Proportion of cancer patients hospitalised for influenza[b] with outcome, N(%)**	**Hazards ratio(HR),[c] 95% CI**	**Excess burden(EB), weighted, per 1000-individuals,[d] 95%CI**
Any post-acute diagnosis[e]	271/1518 (17.9)	50/374 (13.4)	1.08 (0.77-1.51)	9.43 (-30.48-49.35)
Any cardiovascular diagnosis	167/2985 (5.6)	27/637 (4.2)	1.01 (0.64-1.59)	0.34 (-17.26-17.94)
Any neurological diagnosis	153/2818 (5.4)	31/637 (4.9)	0.87 (0.57-1.34)	-6.72 (-25.96-12.52)
Any psychiatric diagnosis	26/3410 (0.8)	7/706 (1.0)	0.61 (0.24-1.56)	-3.13 (-10.14-3.89)
Any autoimmune diagnosis	6/3497 (0.2)	1/723 (0.1)	1.01 (0.10-10.29)	0.02 (-3.52-3.57)
Any respiratory diagnosis	41/3255 (1.3)	13/628 (2.1)	0.52 (0.25-1.08)	-10.07 (-21.92-1.77)
Any renal diagnosis	173/2769 (6.2)	17/620 (2.7)	1.32 (0.78-2.23)	10.93 (-5.55-27.41)
Any gastrointestinal diagnosis	137/2953 (4.6)	16/641 (2.5)	1.59 (0.91-2.80)	14.12 (0.31-27.92)
Any post-acute symptom[f]	193/2940 (6.6)	23/622 (3.7)	1.44 (0.90-2.29)	16.72 (-0.73-34.17)
**Composite post-acute sequelae :**	**Proportion of cancer patients hospitalised for COVID-19[a] with outcome, N(%)**	**Proportion of test-negative cancer patients hospitalized for viral pneumonia[g] with outcome, N(%)**	**Hazards ratio(HR),[a] 95% CI**	**Excess burden(EB), weighted, per 1000-individuals,[b] 95%CI**
Any post-acute diagnosis[e]	273/1528 (17.9)	26/194 (13.4)	1.24 (0.81-1.89)	33.79 (-18.53-86.11)
Any cardiovascular diagnosis	172/3031 (5.7)	18/507 (3.6)	1.23 (0.71-2.12)	8.53 (-9.72-26.79)
Any neurological diagnosis	155/2856 (5.4)	28/490 (5.7)	0.77 (0.49-1.21)	-13.61 (-36.26-9.04)
Any psychiatric diagnosis	26/3459 (0.8)	1/547 (0.2)	-#	-#
Any autoimmune diagnosis	7/3549 (0.2)	1/557 (0.2)	-#	-#
Any respiratory diagnosis	41/3300 (1.2)	6/322 (1.9)	0.71 (0.29-1.76)	-5.39 (-20.81-10.02)
Any renal diagnosis	174/2793 (6.2)	16/498 (3.2)	1.65 (0.94-2.91)	19.87 (2.59-37.16)
Any gastrointestinal diagnosis	140/3000 (4.7)	20/500 (4.0)	0.95 (0.57-1.57)	-1.83 (-20.20-16.53)
Any post-acute symptom[f]	197/2983 (6.6)	30/461 (6.5)	0.84 (0.55-1.29)	-10.55 (-35.39-14.30)
**Composite post-acute sequelae :**	**Proportion of cancer patients hospitalised for COVID-19[a] with outcome, N(%)**	**Proportion of test-negative hospitalized cancer patients[h] with outcome, N(%)**	**Hazards ratio(HR),[a] 95% CI**	**Excess burden(EB), weighted, per 1000-individuals,[b] 95%CI**
Any post-acute diagnosis[e]	273/1528 (17.9)	1022/9267 (11.0)	**1.25 (1.07-1.45)****	31.38 (11.03-51.73)

© 2026 Wee LE et al. *JAMA Network Open*.

Any cardiovascular diagnosis	172/3031 (5.7)	334/13606 (2.5)	**1.48 (1.20-1.82)*** **	17.26 (8.61-25.92)
Any neurological diagnosis	155/2856 (5.4)	451/13330 (3.4)	1.21 (0.99-1.48)	8.89 (0.03-17.75)
Any psychiatric diagnosis	26/3459 (0.8)	67/14177 (0.5)	1.31 (0.79-2.16)	1.64 (-1.39-4.67)
Any autoimmune diagnosis	7/3549 (0.2)	19/14544 (0.1)	1.20 (0.45-3.20)	0.30 (-1.25-1.86)
Any respiratory diagnosis	41/3300 (1.2)	109/13869 (0.8)	1.09 (0.73-1.63)	0.95 (-3.09-5.00)
Any renal diagnosis	174/2793 (6.2)	360/13033 (2.8)	**1.34 (1.09-1.65)** **	14.27 (4.99-23.56)
Any gastrointestinal diagnosis	140/3000 (4.7)	483/12807 (3.8)	1.12 (0.91-1.38)	4.89 (-3.37-13.15)
Any post-acute symptom[f]	197/2983 (6.6)	513/13289 (3.9)	**1.31 (1.09-1.58)** **	14.57 (5.11-24.02)

Abbreviations: CI, confidence interval; HR, hazard ratio; EB, excess burden

[a] COVID-19 hospitalisation was defined as any hospitalisation attributed to COVID-19, reported in the national COVID-19 database. For comparison against influenza hospitalisations, COVID-19 cases hospitalised for influenza within 300 days of COVID-19 test date were additionally excluded.

[b] Influenza hospitalisations were identified using ICD-10 codes in Mediclaims (J09-J11) recorded from 2017-2022.

[c] Competing risks regression taking death as a competing risk, with overlap weights employed. Weighting was based on demographic characteristics (age, sex, ethnicity), socioeconomic status (housing type), comorbidities, prior healthcare utilisation, vaccination status, type of cancer, time from cancer diagnosis to index date.

[d] Excess-burden (EB) of new-incident sequelae per 1,000 individuals at 300 days of follow-up was defined as increase/decrease in incidence rate of new-incident sequelae amongst COVID-19 hospitalisations and comparator group in individuals with cancer

[e] Any post-acute diagnosis was taken as a composite of any cardiovascular/neurological/psychiatric/autoimmune/respiratory/renal/gastrointestinal diagnoses in cancer patients.

[f] Any post-acute symptom was taken as a composite of any individual post-acute symptoms, including cardiovascular signs/symptoms, respiratory signs/symptoms, headache, musculoskeletal pain/stiffness, abdominal/pelvic pain, generalised pain, loss of smell or taste, memory and cognitive impairment, fatigue and malaise.

[g] Defined as contemporaneous test-negatives with a hospitalisation recorded in Mediclaims for viral pneumonia, not otherwise specified (identified using ICD-10 codes J12[x] in Mediclaims, occurring within 14 days of test-negative date).

[h] Defined as contemporaneous test-negatives with any hospitalisation recorded in Mediclaims occurring within 14 days of test-negative date.

*=p<0.05, **=p<0.01, ***=p<0.001. P-values<0.005 (composite outcomes) were taken as being additionally robust to multiple comparisons (Bonferonni)

-#: could not be computed as too few new-incident cases

© 2026 Wee LE et al. *JAMA Network Open*.

eTable 9. Hazards Ratio (HR) of Composite New-Incident Postacute Sequelae in Hospitalized vs Nonhospitalized SARS-CoV-2–Infected Patients With Cancer

**Composite post-acute sequelae :**	**Proportion of SARS-CoV-2 infected hospitalised cancer patients with outcome, N(%)**	**Proportion of SARS-CoV-2 infected non-hospitalised cancer patients with outcome, N(%)**	**Hazards ratio(HR),[a] 95% CI**	**Excess burden(EB), weighted, per 1000-individuals,[b] 95%CI**
Any post-acute diagnosis[c]	273/1528 (17.9)	2047/26107 (7.8)	**1.54 (1.34-1.78)*** **	57.26 (37.81-76.70)
Any cardiovascular diagnosis	172/3031 (5.7)	560/34065 (1.6)	**1.63 (1.34-1.99)*** **	20.69 (12.39-29.00)
Any neurological diagnosis	155/2856 (5.4)	750/33475 (2.2)	**1.44 (1.18-1.75)*** **	15.61 (7.19-24.03)
Any psychiatric diagnosis	26/3459 (0.8)	131/35024 (0.4)	1.47 (0.92-2.36)	2.26 (-0.63-5.14)
Any autoimmune diagnosis	7/3549 (0.2)	47/35602 (0.1)	1.54 (0.64-3.70)	0.69 (-0.82-2.19)
Any respiratory diagnosis	41/3300 (1.2)	228/34571 (0.7)	1.15 (0.79-1.67)	1.51 (-2.31-5.34)
Any renal diagnosis	174/2793 (6.2)	558/33251 (1.7)	**1.67 (1.37-2.03)*** **	22.89 (13.98-31.79)
Any gastrointestinal diagnosis	140/3000 (4.7)	1021/31988 (3.2)	1.16 (0.95-1.41)	6.16 (-1.63-13.95)
Any post-acute symptom[d]	197/2983 (6.6)	980/33334 (2.9)	**1.46 (1.22-1.74)*** **	19.78 (10.68-28.88)

HR > 1 denotes higher risk of a respective composite outcome amongst hospitalized SARS-CoV-2-infected cancer patients versus non-hospitalized SARS-CoV-2-infected cancer patients. Excess burden > 0 denotes excess burden of a respective composite outcome amongst hospitalized SARS-CoV-2-infected cancer patients versus non-hospitalized SARS-CoV-2-infected cancer patients. *=p<0.05, **=p<0.01, ***=p<0.001. P-values<0.005 were taken as being additionally robust to multiple comparisons (Bonferonni). -# denotes that HRs could not be estimated due to too few numbers (≤1 new-incident case in any group, for that specific diagnosis)

Abbreviations: CI, confidence interval; HR, hazard ratio; EB, excess burden

[a] Competing risks regression taking death as a competing risk, with overlap weights employed. For cancer patients: overlap weighted based on demographic characteristics (age, sex, ethnicity), socioeconomic status (housing type), COVID-19 vaccination status, comorbidities, prior healthcare utilisation, prior influenza vaccination, type of cancer, time from cancer diagnosis to index date, on active cancer treatment, prevailing SARS-CoV-2 variant in circulation. Numbers in each subcohort do not add up to the original number of COVID-19 cases because for estimation of risks for each new-incident diagnosis, a sub-cohort of individuals without history of the diagnosis in the past 5 years was constructed.
[b] Excess-burden (EB) of new-incident sequelae per 1,000 individuals at 300 days of follow-up was defined as increase/decrease in incidence rate of new-incident sequelae amongst hospitalized SARS-CoV-2-infected cancer patients versus non-hospitalized SARS-CoV-2-infected cancer patients
[c] Any post-acute diagnosis was taken as a composite of any cardiovascular/neurological/psychiatric/autoimmune/respiratory/renal/gastrointestinal diagnoses in cancer patients.
[d] Any post-acute symptom was taken as a composite of any individual post-acute symptoms, including cardiovascular signs/symptoms, respiratory signs/symptoms, headache, musculoskeletal pain/stiffness, abdominal/pelvic pain, generalised pain, loss of smell or taste, memory and cognitive impairment, fatigue and malaise.
[e] COVID-19 hospitalisation was defined as any hospitalisation attributed to COVID-19, reported in the national COVID-19 database.

© 2026 Wee LE et al. *JAMA Network Open*.

eTable 10. Hazards Ratio (HR) of Postacute Use of Health Care Resources (All-Cause) in SARS-CoV-2–Infected Patients With Cancer and Comparators

**Post-acute healthcare utilisation**	**Proportion of SARS-CoV-2 infected cancer patients with outcome, N(%)**	**Proportion of test-negative cancer patients with outcome, N(%)**	**Hazards ratio(HR),[a] 95% CI**	**Excess burden(EB), weighted, per 1000-individuals,[b] 95%CI**
**Cancer patients with COVID-19 not requiring initial hospitalisation**				
Any post-acute hospitalisation (all-cause)	12755/35685 (35.7)	13225/37551 (35.2)	1.01 (0.98-1.03)	2.25 (-4.68-9.19)
Any post-acute emergency-department visit (all-cause)	4701/35685 (13.2)	4860/37551 (12.9)	0.99 (0.95-1.03)	-0.94 (-5.83-3.94)
**Cancer patients hospitalised for COVID-19[c]**				
Any post-acute hospitalisation (all-cause)	2334/3571 (65.4)	13225/37551 (35.2)	**1.50 (1.43-1.58)*** **	139.94 (123.39-156.49)
Any post-acute emergency-department visit (all-cause)	1403/3571 (39.3)	4860/37551 (12.9)	**1.61 (1.50-1.72)*** **	115.65 (99.24-132.06)
**Post-acute healthcare utilisation**	**Proportion of cancer patients hospitalised for COVID-19[c] with outcome, N(%)**	**Proportion of cancer patients hospitalised for influenza[d] with outcome, N(%)**	**Hazards ratio(HR),[e] 95% CI**	**Excess burden(EB), weighted, per 1000-individuals,[f] 95%CI**
Any post-acute hospitalisation (all-cause)	2292/3513 (65.2)	376/721 (52.1)	**1.16 (1.03-1.31)** **	46.57 (6.71-86.42)
Any post-acute emergency-department visit (all-cause)	1372/3513 (39.1)	160/721 (22.2)	**1.36 (1.14-1.62)*** **	63.52 (28.41-98.64)

Abbreviations: CI, confidence interval; HR, hazard ratio; EB, excess burden

[a] Competing risks regression taking death as a competing risk, with overlap weights employed. For cancer patients: overlap weighted based on demographic characteristics (age, sex, ethnicity), socioeconomic status (housing type), COVID-19 vaccination status, comorbidities, prior healthcare utilisation, prior influenza vaccination, type of cancer, time from cancer diagnosis to index date, on active cancer treatment, prevailing SARS-CoV-2 variant in circulation. Numbers in each subcohort do not add up to the original number of COVID-19 cases/test-negatives because for estimation of risks for each new-incident diagnosis, a sub-cohort of individuals without history of the diagnosis in the past 5 years was constructed.

[b] Excess-burden (EB) of post-acute healthcare utilisation per 1,000 individuals at 300 days of follow-up was defined as increase/decrease in incidence rate of post-acute healthcare utilisation amongst COVID-19 cases and test-negatives in cancer patients

[c] COVID-19 hospitalisation was defined as any hospitalisation attributed to COVID-19, reported in the national COVID-19 database.

[d] Influenza hospitalisations were identified using ICD-10 codes in Mediclaims (J09-J11) recorded from 2017-2022.

[e] Competing risks regression taking death as a competing risk, with overlap weights employed. For cancer patients: overlap weighted based on demographic characteristics (age, sex, ethnicity), socioeconomic status (housing type), comorbidities, prior healthcare utilisation, vaccination status, type of cancer, time from cancer diagnosis to index date. Numbers do not add up to the original number of COVID-19 hospitalisations because COVID-19 cases hospitalised for influenza within 300 days of COVID-19 test date were additionally excluded.

[f] Excess-burden (EB) of new-incident sequelae per 1,000 individuals at 300 days of follow-up was defined as increase/decrease in incidence rate of new-incident sequelae amongst COVID-19 hospitalisations and influenza hospitalisations in individuals with cancer

*=p<0.05, **=p<0.01, ***=p<0.001.

© 2026 Wee LE et al. *JAMA Network Open*.

eTable 11. Hazards Ratio (HR) of Negative Outcome Controls (Atopic Dermatitis, Limb Injuries)

Outcomes	Proportion of SARS-CoV-2 infected cancer patients with outcome, N(%)	Proportion of test-negative cancer patients with outcome, N(%)	Hazards ratio(HR),[a] 95% CI	Excess burden(EB), weighted, per 1000-individuals,[b] 95%CI
Atopic dermatitis	18/39193 (0.0)	23/37502 (0.1)	0.73 (0.39-1.36)	-0.17 (-0.49-0.16)
Injury to upper limbs	213/38832 (0.5)	208/37211 (0.6)	0.94 (0.77-1.14)	-0.36 (-1.41-0.69)
Injury to lower limbs	269/38664 (0.7)	226/37072 (0.6)	1.05 (0.88-1.26)	0.34 (-0.81-1.49)

HR > 1 denotes higher risk of a respective negative-outcome control amongst COVID-19 cases and test-negatives in individuals with cancer. Excess burden > 0 denotes excess burden of a respective negative-outcome control amongst COVID-19 cases and test-negatives in individuals with cancer.
Abbreviations: CI, confidence interval; HR, hazard ratio; EB, excess burden

[a] Competing risks regression taking death as a competing risk, with overlap weights employed. For cancer patients: overlap weighted based on demographic characteristics (age, sex, ethnicity), socioeconomic status (housing type), COVID-19 vaccination status, comorbidities, prior healthcare utilisation, influenza vaccination, type of cancer, time from cancer diagnosis to index date, on active cancer treatment, prevailing SARS-CoV-2 variant in circulation. Numbers in each subcohort do not add up to the original number of COVID-19 cases/test-negatives because for estimation of risks for each new-incident diagnosis, a sub-cohort of individuals without history of the diagnosis in the past 5 years was constructed.
[b] Excess-burden (EB) of new-incident sequelae per 1,000 individuals at 300 days of follow-up was defined as increase/decrease in incidence rate of new-incident sequelae amongst COVID-19 cases and test-negatives in individuals with cancer.

© 2026 Wee LE et al. *JAMA Network Open*.

eFigure 1. Distribution of Test Date in SARS-CoV-2–Infected and Noninfected Patients With Cancer

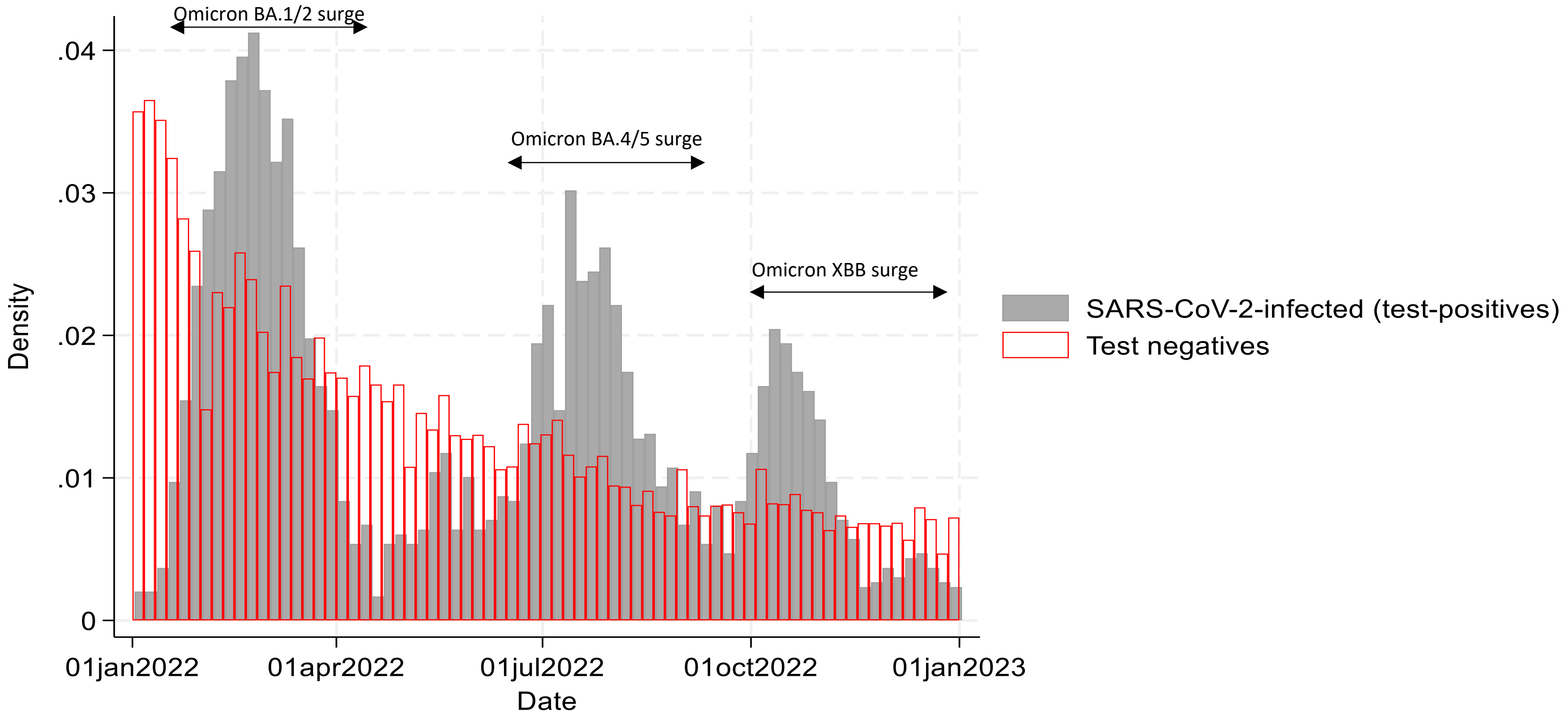

© 2026 Wee LE et al. *JAMA Network Open*.

eFigure 2. Distribution of Test Date in SARS-CoV-2–Infected and Noninfected Patients With Cancer (After Matching by Test Date)

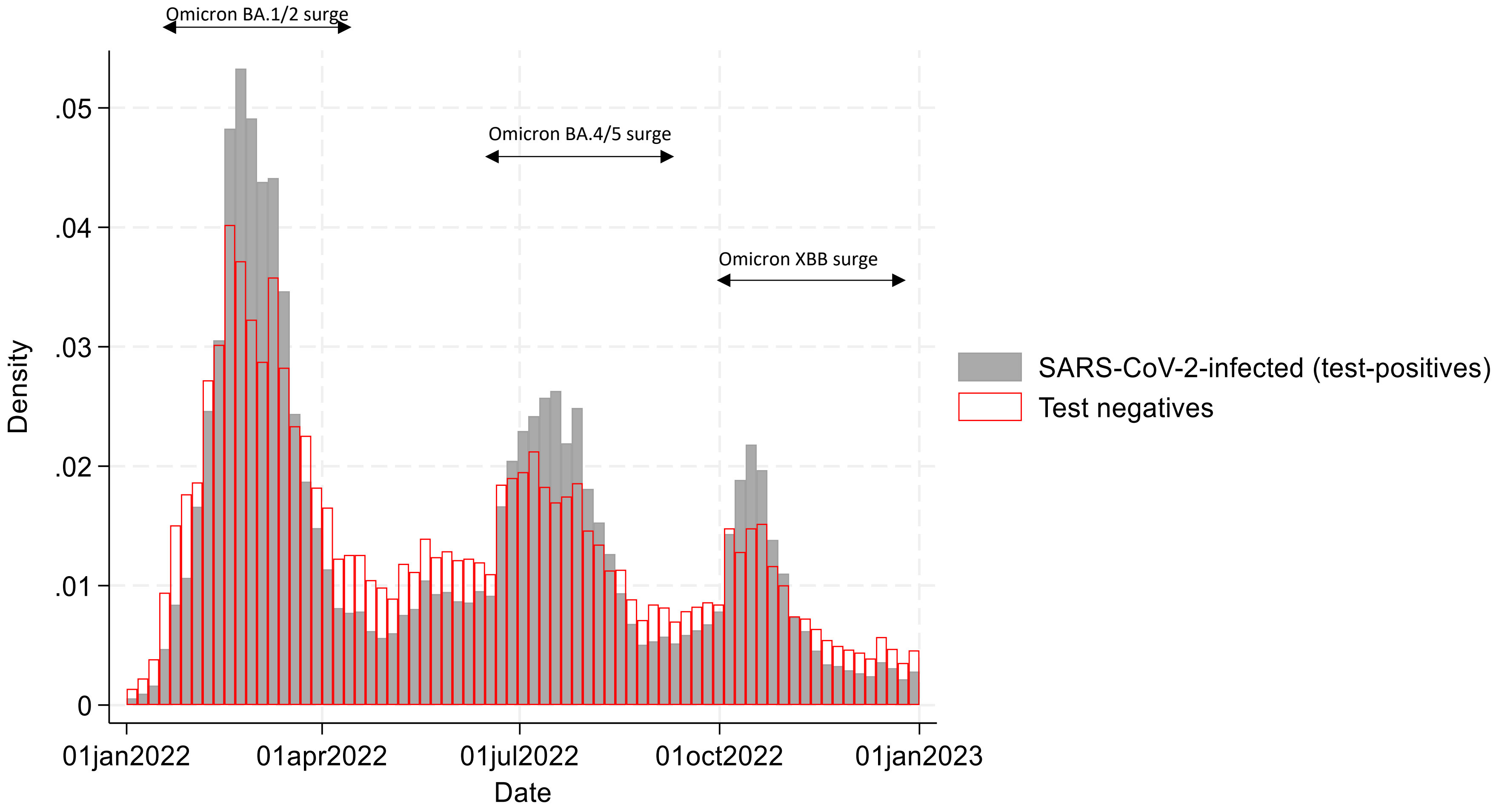

© 2026 Wee LE et al. *JAMA Network Open*.

eAppendix. List of *ICD-10* Codes Used for Diagnoses of Interest

Note: where x is indicated in superscript, it indicates that all subcodes under that particular ICD-10 code are additionally included.

**Pre-specified cardiovascular outcomes**<br><br>**(subcategories:**<br><br>**Dysrhythmia; inflammatory heart diseases; other heart diseases; thrombotic diseases)**	**Outcome**	**ICD 10 Code**	**Description**
	**Dysrhythmia**		
	Atrial fibrillation	I48^{x}	Atrial fibrillation and flutter
	Sinus tachycardia	I47^{x}	Paroxysmal tachycardia
		R00.0	Tachycardia, unspecified
	Sinus bradycardia	R00.1	Bradycardia, unspecified
	Other arrhythmias	I44^{x}	Atrioventricular and left bundle-branch block
		R00.2	Palpitations
		R00.8	Other abnormalities of heart beat
		R00.9	Unspecified abnormalities of heart beat
		I45^{x}	Other conduction disorders
	**Inflammatory heart disease**		
		I49^{x}	Other cardiac arrhythmias
	Pericarditis	I30^{x}	Acute pericarditis

© 2026 Wee LE et al. *JAMA Network Open*.

	B33.23	Viral pericarditis
Myocarditis	I51.4	Myocarditis, unspecified
	B33.20	Viral carditis, unspecified
	B33.21	Viral endocarditis
	B33.22	Viral myocarditis
	B33.24	Viral cardiomyopathy
	I40[x]	Acute myocarditis
**Ischemic heart disease**		
Myocardial infarction	I21[x]	Acute myocardial infarction
	I22[x]	Subsequent ST elevation (STEMI) and non-ST elevation (NSTEMI) myocardial infarction
Acute coronary disease	I24[x]	Other acute ischemic heart diseases
	I25.10	Atherosclerotic heart disease of native coronary artery without angina pectoris
	I25.11[x]	Atherosclerotic heart disease of native coronary artery with angina pectoris
Ischemic cardiomyopathy	I25.5	Ischemic cardiomyopathy
Angina	I20[x]	Angina pectoris
**Other heart conditions**		
Heart failure	I50.1	Left ventricular failure, unspecified
	I50.21	Acute systolic (congestive) heart failure

© 2026 Wee LE et al. *JAMA Network Open*.

	150.23	Acute on chronic systolic (congestive) heart failure
	150.31	Acute diastolic (congestive) heart failure
	I50.33	Acute on chronic diastolic (congestive) heart failure
	I50.41	Acute combined systolic (congestive) and diastolic (congestive) heart failure
	I50.43	Acute on chronic combined systolic (congestive) and diastolic (congestive) heart failure
	I50.811	Acute right heart failure
	I50.813	Acute on chronic right heart failure
Cardiomyopathy	I42[x]	Cardiomyopathy
Cardiac arrest	I46[x]	Cardiac arrest
Cardiogenic shock	R57.0	Cardiogenic shock
**Thrombotic conditions**		
Pulmonary embolism	I26[x]	Pulmonary embolism
Deep venous thrombosis	I80.1[x]	Phlebitis and thrombophlebitis of femoral vein
	I80.2[x]	Phlebitis and thrombophlebitis of other and unspecified deep vessels of lower extremities
	I81	Portal vein thrombosis
	I82 [x]	Other venous embolism and thrombosis
	I67.6	Nonpyogenic thrombosis of intracranial venous system
Superficial venous thrombosis	I80.0[x]	Phlebitis and thrombophlebitis of superficial vessels of lower extremities

© 2026 Wee LE et al. *JAMA Network Open*.

		I80.3	Phlebitis and thrombophlebitis of lower extremities, unspecified
		I80.8	Phlebitis and thrombophlebitis of other sites
		I80.9	Phlebitis and thrombophlebitis of unspecified site
	Arterial thromboses	I74[x]	Arterial embolism and thrombosis
**Pre-specified neurological outcomes**<br><br>**(subcategories:**<br><br>**Cerebrovascular disease; peripheral neuropathies; episodic disorders; extrapyramidal and movement disorders; sensory disorders; other neurological disorders; memory problems)**	Outcome	**ICD 10 Code**	**Description**
	**Cerebrovascular disease**		
	Ischemic stroke	I63[x]	Cerebral infarction
		G46[x]	Vascular syndromes of brain in cerebrovascular diseases
	Transient ischemic attack (TIA)	G45[x]	Transient cerebral ischaemic attacks and related syndromes
		I65[x]	Occlusion and stenosis of precerebral arteries not resulting in cerebral infarction

© 2026 Wee LE et al. *JAMA Network Open*.

		I66[x]	Occlusion and stenosis of cerebral arteries not resulting in cerebral infarction
	Haemorrhagic stroke	I60[x]	Nontraumatic subarachnoid hemorrhage
		I61[x]	Intracerebral hemorrhage
		I62[x]	Other non-traumatic intracranial hemorrhage
	Cerebral infarction	I63[x]	Cerebral infarction
	**Peripheral neuropathies**		
	Peripheral neuropathy	G61.1	Serum neuropathy
		G61.8[x]	Other inflammatory polyneuropathies
		G61.9	Inflammatory polyneuropathy, unspecified
		G62[x]	Other and unspecified polyneuropathies
		G56[x]	Mononeuropathies of upper limbs
		G57[x]	Mononeuropathies of lower limbs
	Paresthesia	R20.1	Hypoesthesia of skin
		R20.2	Paraesthesia of skin
		R20.3	Hyperesthesia
	Dysautonomia	G90.0[x]	Idiopathic peripheral autonomic neuropathy
		G90.3	Multi-system degeneration of the autonomic nervous system
		G90.4	Autonomic dysreflexia
		G90.8[x]	Other disorders of autonomic nervous system

© 2026 Wee LE et al. *JAMA Network Open*.

		G90.9	Disorder of the autonomic nervous system
	Bell's palsy	G51[x]	Facial nerve disorders
	**Episodic disorders**		
	Migraine	G43[x]	Migraine
	Headache disorders	G44.0[x]	Cluster headaches and other trigeminal autonomic cephalgias
		G44.1[x]	Vascular headache, not elsewhere classified
		G44.2[x]	Tension-type headache
		G44.5[x]	Complicated headache syndromes
		G44.8[x]	Other unspecified headache syndromes
	Epilepsy and seizures	G40[x]	Epilepsy and seizures
	**Extrapyramidal and movement disorders**		
	Abnormal involuntary movements	R25[x]	Other abnormal involuntary movements
	Tremor	G25.0	Essential tremor
		G25.2	Other specified forms of tremor
	Dystonia	G24.3	Spasmodic torticolis
		G24.4	Idiopathic orofacial dystonia
		G24.5	Blepharospasm
		G24.8	Other dystonia
		G24.9	Dystonia, unspecified

© 2026 Wee LE et al. *JAMA Network Open*.

	Other extrapyramidal and movement disorders	G25.3	Myoclonus
		G25.5	Other chorea
		G25.89	Other specified extrapyramidal and movement disorders
		G25.9	Extrapyramidal and movement disorder, unspecified
	Tic disorders	F95[x]	Tic disorder
		F98.4	Stereotyped movement disorders
	Parkinson’s disease	G20[x]	Parkinson’s disease
	**Sensory disorders**		
	Hearing abnormalities or tinnitus	H90[x]	Conductive and sensorineural hearing loss
		H91[x]	Other and unspecified hearing loss
		H93.1[x]	Tinnitus
		H93.2[x]	Other abnormal auditory perceptions
		H93.3[x]	Disorders of acoustic nerve
		H93.9[x]	Unspecified disorder of ear
	Visual abnormalities	H53[x]	Visual disturbances
		H54[x]	Blindness and low vision
		H34[x]	Retinal vascular occlusions
		H35.6[x]	Retinal hemorrhage

© 2026 Wee LE et al. *JAMA Network Open*.

Loss of smell or taste	R43[x]	Disturbances of smell and taste
**Other neurological disorders**		
Dizziness	R42 [x]	Dizziness and giddiness
Sleep disorders	G47.0[x]	Insomnia
	G47.1[x]	Hypersomnia
	G47.30	Sleep apnea, unspecified
	G47.36	Sleep related hypoventilation in conditions classified elsewhere
	G47.39	Other sleep apnea
	G47.4[x]	Narcolepsy and cataplexy
	G47.5[x]	Parasomnia
	G47.6[x]	Sleep-related movement disorders
	F51[x]	Sleep disorders not due to a substance or known physiological condition
Somnolence, malaise and fatigue	R53.1	Weakness
	R53.8[x]	Other malaise and fatigue
	G93.3[x]	Post-viral fatigue syndrome
Guillain-Barre syndrome	G61.0	Guillain-Barre syndrome
Encephalitis or encephalopathy	G04.0[x]	Acute disseminated encephalitis and encephalomyelitis
	G04.3[x]	Acute necrotizing hemorrhagic encephalopathy
	G04.81	Other encephalitis and encephalomyelitis

© 2026 Wee LE et al. *JAMA Network Open*.

	G04.90	Encephalitis and encephalomyelitis, unspecified
Transverse myelitis	G35[x]	Multiple sclerosis
	G36.0	Neuromyelitis optica
	G37.3	Acute transverse myelitis in demyelinating disease of central nervous system
	G04.82	Acute flaccid myelitis
	G04.89	Other myelitis
	G04.91	Myelitis, unspecified
**Memory and cognitive problems**		
Memory problems	R41.1	Anterograde amnesia
	R41.2	Retrograde amnesia
	R41.3	Other amnesia
	R41.84	Other specified cognitive deficit
	R41.85	Anosognosia
	R41.89	Other symptoms and signs involving cognitive functions and awareness
	R41.9	Unspecified symptoms and signs involving cognitive functions and awareness
	G31.0[x]	Frontotemporal dementia
	G31.83	Neurocognitive disorder with Lewy bodies
	G31.84	Mild cognitive impairment of uncertain or unknown etiology

© 2026 Wee LE et al. *JAMA Network Open*.

		G31.85	Corticobasal degeneration
		G31.89	Other specified degenerative diseases of nervous system
		G31.9	Degenerative disease of nervous system, unspecified
		F01^{x}	Vascular dementia
		F02^{x}	Dementia in other diseases classified elsewhere
		F03^{x}	Unspecified dementia
		F44^{x}	Dissociative and conversion disorders
		G45.4	Transient global amnesia
	Alzheimer’s disease	G30^{x}	Alzheimer’s disease
**Pre-specified psychiatric outcomes**<br><br>**(subcategories:**<br><br>**Mood disorders; stress/adjustment disorders and anxiety disorders; psychotic disorders)**	**Outcome**	**ICD 10 Code**	**Description**
	**Mood disorders**		
	Major depressive disorder	F32^{x}	Major depressive disorder, single episode
		F33^{x}	Major depressive disorder, recurrent
		R45.84	Anhedonia
		R45.851	Suicidal ideation

© 2026 Wee LE et al. *JAMA Network Open*.

Other mood disorders	F30^{x}	Manic episode
	F31^{x}	Bipolar disorder
	F34^{x}	Persistent mood [affective] disorders
**Stress/adjustment and anxiety disorders**		
Stress/adjustment disorders	F43.0	Acute stress reaction
	F43.1^{x}	Post-traumatic stress disorder
	F43.2^{x}	Adjustment disorder
Anxiety disorders	F41^{x}	Other anxiety disorders
	F42^{x}	Obsessive-compulsive disorder
**Psychotic disorders**		
Psychotic disorders	F20^{x}	Schizophrenia
	F21^{x}	Schizotypal disorder
	F22^{x}	Delusional disorders
	F23^{x}	Brief psychotic disorder
	F24^{x}	Shared psychotic disorder
	F25^{x}	Schizoaffective disorder
	F28^{x}	Other psychotic disorder not due to a substance or known physiological condition
	F29^{x}	Unspecified psychosis not due to a substance or known physiological condition

© 2026 Wee LE et al. *JAMA Network Open*.

Pre-specified autoimmune outcomes (subcategories: systemic lupus and other connective tissue disorders, vasculitis)

Outcome	ICD 10 Code	Description
**Systemic lupus and other connective tissue disorders**		
Systemic lupus	M32[x]	Systemic lupus erythematosus
Other connective tissue disorders	M35.0[x]	Sjogren syndrome
	M34[x]	Systemic sclerosis
	M33[x]	Dermatopolymyositis
	M35.1	Mixed connective tissue disease, other overlap syndromes
	M35.2	Behcet's disease
	M35.3	Polymyalgia rheumatica
	M45[x]	Ankylosing spondylitis
	M46[x]	Other inflammatory spondylopathies
	M02.3[x]	Reiter's disease
	M02.1[x]	Postdysenteric arthropathy
**Vasculitis**		
	M30.0	Polyarteritis nodosa
	M30.1	Polyarteritis with lung involvement [Churg-Strauss]
	M30.2	Juvenile polyarteritis
	M30.8	Other conditions related to polyarteritis nodosa

© 2026 Wee LE et al. *JAMA Network Open*.

		M31[x]	Other necrotizing vasculopathies (including Wegener's granulomatosis, Aortic arch syndrome [Takayasu], Giant cell arteritis)
		L95[x]	Vasculitis limited to skin, not elsewhere classified
		I77.82	Antineutrophilic cytoplasmic antibody [ANCA] vasculitis
		I79.1	Aortitis in diseases classified elsewhere (isolated aortitis)
	**Outcome**	**ICD 10 Code**	**Description**
**Pre-specified respiratory outcomes (subcategories: asthma, COPD)**	**Outcome**	**ICD 10 Code**	**Description**
	**Chronic-obstructive pulmonary disease**		
		J41[x]	Simple and mucopurulent chronic bronchitis
		J42	Unspecified chronic bronchitis
		J43[x]	Emphysema
		J44[x]	Other chronic obstructive pulmonary disease
	**Asthma**	J45[x]	Asthma
	Bronchiectasis	J47[x]	Bronchiectasis
	Pulmonary fibrosis	J84.1[x]	Other interstitial pulmonary diseases with fibrosis
		J84.89	Other specified interstitial pulmonary diseases
		J84.9	Interstitial pulmonary disease, unspecified
	**Outcome**	**ICD 10 Code**	**Description**

© 2026 Wee LE et al. *JAMA Network Open*.

**Pre-specified renal outcomes**	Acute kidney failure	N17[x]	Acute kidney failure
	Chronic kidney disease	N18[x]	Chronic kidney disease (CKD)
	End stage renal failure	I12.0	Hypertensive kidney disease with kidney failure
		I13.11	Hypertensive heart and kidney disease with kidney failure
		N19	Unspecified kidney failure
		Z49[x]	Encounter for care involving renal dialysis
		Z99.2	Dependence on renal dialysis
		Z94.0	Kidney transplant status
	**Outcome**	**ICD 10 Code**	**Description**
**Pre-specified gastrointestinal outcomes** **(subcategories: appendicitis, inflammatory bowel disease, gastritis, biliary tract disease, hepatitis)**	**Appendicitis**	K35[x]	Acute appendicitis
	Celiac disease	K90.0	Celiac disease
	**Inflammatory bowel disease**	K50[x]	Crohn's disease
		K51[x]	Ulcerative colitis
		K52[x]	Other and unspecified noninfective gastroenteritis and colitis
	**Gastritis/peptic ulcer disease**	K29[x]	Gastritis and duodenitis
		K25[x]	Gastric ulcer

© 2026 Wee LE et al. *JAMA Network Open*.

		K26^{x}	Duodenal ulcer
		K27^{x}	Peptic ulcer, site unspecified
		K28^{x}	Gastrojejunal ulcer
	Irritable bowel syndrome (IBS)	K58^{x}	Irritable bowel syndrome
	**Biliary tract disease**	K80^{x}	Cholelithiasis
		K83^{x}	Other diseases of biliary tract
	**Hepatitis and cirrhosis**	K73^{x}	Chronic hepatitis, not elsewhere classified
		K75.2	Nonspecific reactive hepatitis
		K75.3	Granulomatous hepatitis, not elsewhere classified
		K75.4	Autoimmune hepatitis
		K75.8^{x}	Other specified inflammatory liver diseases
		K71^{x}	Toxic liver disease
		K74^{x}	Fibrosis and cirrhosis of liver

List of ICD-10 codes used for outcomes of interest, symptom-based diagnoses

© 2026 Wee LE et al. *JAMA Network Open*.

Outcome	ICD 10 Code	Description
Cardiovascular signs and symptoms	R00[x]	Abnormalities of heart beat
	R01[x]	Cardiac murmurs and other cardiac sounds
	R03[x]	Abnormal blood-pressure reading, without diagnosis
	R23.0	Cyanosis
	R07.1	Chest pain on breathing
	R07.2	Precordial pain
	R07.8[x]	Other chest pain
Respiratory signs and symptoms	R04[x]	Hemorrhage from respiratory passages
	R05[x]	Cough
	R06[x]	Abnormalities of breathing
Headache	R51[x]	Headache
Musculoskeletal pain/stiffness	M25.5[x]	Pain in joint
	M79[x]	Other and unspecified soft tissue disorders, not elsewhere classified (including fibromyalgia)
	M25.6[x]	Stiffness of joint, not elsewhere classified
	M54[x]	Dorsalgia
Abdominal and pelvic pain	R10[x]	Abdominal and pelvic pain
	G43.D[x]	Abdominal migraine
Generalised pain	G89[x]	Pain, not elsewhere classified
	R52[x]	Pain, unspecified

© 2026 Wee LE et al. *JAMA Network Open*.

Loss of smell or taste	R43[x]	Disturbances of smell and taste
Memory and cognitive impairment	R41.1	Anterograde amnesia
	R41.2	Retrograde amnesia
	R41.3	Other amnesia
	R41.84	Other specified cognitive deficit
	R41.85	Anosognosia
	R41.89	Other symptoms and signs involving cognitive functions and awareness
	R41.9	Unspecified symptoms and signs involving cognitive functions and awareness
Fatigue and malaise	R53.1	Weakness
	R53.8[x]	Other malaise and fatigue
	G93.3[x]	Post-viral fatigue syndrome
	M62.81	Muscle weakness, generalized

List of ICD-10 codes, negative outcome controls

© 2026 Wee LE et al. *JAMA Network Open*.

Outcome	Description	ICD 10 Code
**Lower limb injury**		
Hip injuries	S70^x	Superficial injury of hip and thigh
	S71^x	Open wound of hip and thigh
	S72^x(including S72.3, fracture of shaft of femur; S72.0, fracture of head and neck of femur; S721, pertrochanteric fracture; S72.2, subtrochanteric fracture of femur, S72.8, other fracture of femur; S72.9, unspecified fracture of femur; S72.4, fracture of lower end of femur)	Hip fracture
	S73^x	Dislocation and sprain of joint and ligaments of hip
	S74^x	Injury of nerves at hip and thigh level
	S75^x	Injury of blood vessels at hip and thigh level
	S76^x	Injury of muscle, fascia and tendon at hip and thigh level
	S77^x	Crushing injury of hip and thigh
	S78^x	Traumatic amputation of hip and thigh
	S79^x	Other and unspecified injuries of hip and thigh
Lower leg injuries	S80x	Superficial injury of knee and lower leg
	S81x	Open wound of knee and lower leg
	S82^x (S82.3, fracture of lower end of tibia; S82.6, fracture of lateral malleolus; S82.8, other fractures of lower leg; S82.9, unspecified fracture of lower leg; S82.4, fracture of shaft of fibula; S82.5, fracture of medial malleolus; S82.0, fracture of patella, S82.1, fracture of upper end of tibia; S82.2, fracture of shaft of tibia)	Fracture of lower leg
	S83^x	Dislocation and sprain of knee and lower leg
	S84^x	Injury of nerves at knee and lower leg level
	S85^x	Injury of blood vessels at knee and lower leg level
	S86^x	Injury of muscle, fascia and tendon at knee and lower leg level

© 2026 Wee LE et al. *JAMA Network Open*.

	S87[x]	Crushing injury of knee and lower leg level
	S88[x]	Traumatic amputation of knee and lower leg level
	S89[x]	Other and unspecified injuries of knee and lower leg level
Foot injuries	S90[x]	Superficial injury of foot
	S91[x]	Open wound of foot
	S92[x] (including S92.0, fracture of calcaneus; S92.3, fracture of metatarsal bone; S92.4, fracture of great toe; S92.5, fracture of lesser toe; S92.1, fracture of talus, S92.2, fracture of other and unspecified tarsal bones, S92.8, other fracture of foot except ankle, S92.9, unspecified fracture of foot and toe)	Fracture of foot/toe
	S93[x]	Dislocation and sprain of foot
	S94[x]	Injury of nerves at foot level
	S95[x]	Injury of blood vessels at foot level
	S96[x]	Injury of muscle, fascia and tendon at foot level
	S97[x]	Crushing injury of foot
	S98[x]	Traumatic amputation of foot
	S99[x]	Other and unspecified injuries of foot
**Upper limb injury**		
Injuries to wrist, hand and fingers	S60[x]	Superficial injury of wrist, hand and fingers
	S61[x]	Open wound of wrist, hand and fingers
	S62[x] (including S62.1, fracture of other and unspecified carpal bones; S62.2, fracture of first metacarpal bone, S62.5, fracture of thumb, S62.6, fracture of other and unspecified fingers, S62.9, unspecified fracture of wrist and hand, S62.0, fracture of scaphoid bone; S62.3, fracture of other and unspecified metacarpal bone)	Fracture at wrist and hand level

© 2026 Wee LE et al. *JAMA Network Open*.

	S63[x]	Dislocation and sprain of wrist, hand and fingers
	S64[x]	Injury of nerves at wrist and hand level
	S65[x]	Injury of blood vessels at wrist and hand level
	S66[x]	Injury of muscle, fascia and tendon at wrist and hand level
	S67[x]	Crushing injury of wrist, hand and fingers
	S68[x]	Traumatic amputation of wrist, hand and fingers
	S69[x]	Other and unspecified injuries of wrist, hand and fingers
Elbow and forearm injuries	S50[x]	Superficial injury of forearm
	S51[x]	Open wound of forearm
	S52[x] (including S52.0, fracture of upper end of ulna; S52.2, fracture of shaft of ulna; S52.3, fracture of shaft of radius; S52.5, fracture of lower end of radius, S52.9, unspecified fracture of forearm, S52.1, fracture of upper end of radius, S52.6, fracture of lower end of ulna)	Fracture of forearm
	S53[x]	Dislocation and sprain of forearm
	S54[x]	Injury of nerves at forearm level
	S55[x]	Injury of blood vessels at forearm level
	S56[x]	Injury of muscle, fascia and tendon at forearm level
	S57[x]	Crushing injury of forearm
	S58[x]	Traumatic amputation of forearm
	S59[x]	Other and unspecified injuries of forearm
Shoulder injuries	S40[x]	Superficial injury of shoulder and upper arm
	S41[x]	Open wound of shoulder and upper arm

© 2026 Wee LE et al. *JAMA Network Open*.

	S42[x] (including S42.1, fracture of scapula; S42.0, fracture of clavicle; S42.2, fracture of upper end of humerus, S42.9, fracture of shoulder girdle, S42.4, fracture of lower end of humerus)	Fracture of shoulder and upper arm
	S43[x]	Dislocation and sprain of shoulder
	S44[x]	Injury of nerves at shoulder and upper arm level
	S45[x]	Injury of blood vessels at shoulder and upper arm level
	S46[x]	Injury of muscle, fascia and tendon at shoulder and upper arm level
	S47[x]	Crushing injury of shoulder and upper arm
	S48[x]	Traumatic amputation of shoulder and upper arm level
	S49[x]	Other and unspecified injuries of shoulder and upper arm
**Atopic dermatitis**	L20[x]	Atopic dermatitis

© 2026 Wee LE et al. *JAMA Network Open*.
