# Supplementary material for: Postacute Sequelae Following Omicron COVID-19 in Patients With Cancer
Source: JAMA Netw Open. 2026 Mar 31;9(3):e264037. doi: 10.1001/jamanetworkopen.2026.4037 (PMC13040405; doi:10.1001/jamanetworkopen.2026.4037)
Supplement: Supplement 2. — Data Sharing Statement [file jamanetwopen-e264037-s002.pdf]

Data Sharing Statement

Wee. Postacute Sequelae Following Omicron COVID-19 in Patients With Cancer. *JAMA Netw Open*. Published March 31, 2026. doi:10.1001/jamanetworkopen.2026.4037

Data
Data available: No

Additional Information
Explanation for why data not available: Data is not available due to personal data protection requirements. Deidentified data can be made available, subject to approval by the Ministry of Health, Singapore. All enquiries to be directed to the corresponding author.
